# Familial patterns of immune dysregulation in CVID: insights from B- and T-cell phenotyping and antibody profiling

**DOI:** 10.3389/fimmu.2026.1741900

**Published:** 2026-03-13

**Authors:** Suzanne E. T. Comans, Evelien G. G. Sprenkeler, Mischa H. Koenen, Elles Simonetti, Bram Van Cranenbroek, Esther Van Rijssen, Riet Strik-Albers, Hans J. P. M. Koenen, Jacques J. M. van Dongen, Lilly M. Verhagen, Marien I. de Jonge, Stefanie S. V. Henriet

**Affiliations:** 1 Department of Laboratory Medicine, Laboratory of Medical Immunology, Radboud Community for Infectious Diseases, Radboud university medical center, Nijmegen, Netherlands; 2 Department of Pediatrics, Erasmus Medical Center, Rotterdam, Netherlands; 3 Department of Pediatric Infectious Diseases and Immunology, Amalia Children’s Hospital, Radboud university medical center, Nijmegen, Netherlands; 4 Cytometry Service, NUCLEUS; Department of Medicine, University of Salamanca (Universidad de Salamanca), Salamanca, Spain; 5 Translational and Clinical Research Program, Cancer Research Center (IBMCC, CSIC - University of Salamanca), Salamanca, Spain; 6 Department of Immunology, Leiden University Medical Center, Leiden, Netherlands

**Keywords:** antibodies, common variable immunodeficiency (CVID), familial pattern, immunophenotyping, mucosal immunology, primary immunodeficiency

## Abstract

**Introduction:**

Common Variable Immunodeficiency (CVID) is a heterogeneous immune disorder characterized by a broad range of clinical manifestations. Its etiology is not yet fully understood. To gain insights into the immunological background of CVID in patients without a clearly defined genetic cause, we employed a genealogical research approach that included family members. This strategy aims to provide a more comprehensive understanding of immune dysregulation in CVID, of potential hereditary patterns, and subclinical immunological traits within families.

**Methods:**

Fifty-eight participants from nine families were included: Five families with a negative family history for CVID (FH-) and four families with a positive family history for CVID (FH+). Screening for known CVID-associated genes was negative in all cases. The non-affected family members completed a questionnaire covering medical history relevant to primary immunodeficiency. Flow cytometry was used to analyze T- and B-cell subsets in peripheral blood, while mucosal and systemic IgA and IgG levels were measured using a multiplex immunoassay.

**Results:**

Immune aberrancies in CVID patients were observed in the B- and T-cell compartments. In both FH− and FH+ family members, symptoms suggestive of Primary Immunodeficiency (PID) were present in 32.1 to 61.5% (p = 0.29). B-cell subsets were 5- to 10-fold reduced compared with the 5th percentile of age specific reference values. A combination of T-cell and B-cell reductions was observed in eight of the nine families in a non-affected member. Serum and mucosal IgG and IgA levels in FH− families did not differ significantly from FH+ families. There were no significant correlations between systemic and mucosal IgA levels in non-affected family members from either FH- or FH+ families.

**Conclusion:**

Overall, our findings show that B- and T-cell aberrancies are present not only in CVID patients but also in non-affected family members, irrespective of family history. Systemic IgA does not reflect mucosal IgA, and systemic IgG replacement therapy does not restore mucosal antibody levels, highlighting compartmentalized immune regulation.

## Introduction

Common Variable Immunodeficiency (CVID) is one of the most prevalent primary immunodeficiencies in children and adults, with an estimated prevalence of around 1:20,000 to 1:50,000 live births (1). CVID is a heterogeneous disorder characterized by impaired B-cell maturation and defective production of specific immunoglobulins (1). Clinically, CVID can present with a broad range of disease manifestations, including recurrent upper and lower respiratory tract infections, gastrointestinal infections, and bronchiectasis. Additionally, affected individuals may develop associated conditions such as interstitial lung disease, lymphoproliferation, autoimmunity, granulomas, enteropathy, and have an increased risk of developing malignancies (2–5).

Despite advances in the understanding of CVID, its etiology remains incompletely understood. Genetic studies suggest that potential pathogenic variants can be identified in approximately 10–20% of CVID cases, depending on the cohort studied (6–8). The variability can be partly attributed to differences in genetic background, including the presence or absence of consanguinity (9). While monogenic causes of CVID are relatively rare, multiple genetic loci have been proposed as putative risk alleles or disease modifiers. For instance, variants in *TNFRSF13B* (TACI), present in 8-10% of CVID cases, are also found in healthy individuals, suggesting a role as risk factors rather than a direct pathogenic variant (2). The broad range of implicated loci underscores the genetic heterogeneity and clinical variability characteristic of CVID. In addition to genetic factors, recent studies have highlighted the role of epigenetic mechanisms such as altered transcription factor expression, DNA methylation, histone modifications, chromatin modulation, and noncoding RNAs in disease pathogenesis (10).

CVID is diagnosed based on a set of established criteria provided by the European Society of Immunodeficiencies (ESID). These criteria define CVID based on a combination of clinical criteria, including increased susceptibility to infections, autoimmunity, granulomatous disease, and/or unexplained polyclonal lymphoproliferation, hypogammaglobulinemia (decrease of IgG and IgA, with or without low IgM levels), and poor vaccine response and/or reduced switched memory B-cells. The diagnosis should be established after the 4^th^ year of life (but symptoms may be present before), and there should not be evidence of profound T-cell deficiency (11, 12). A family history of antibody deficiency is one of the clinical criteria in the ESID definition, highlighting the importance of comprehensive family assessments in affected individuals. However, subclinical presentation might go unrecognized and thus might be important to investigate.

While conventional B-cell subset analysis often fails to capture the full spectrum of B-cell abnormalities in CVID, advances in high-dimensional flow cytometry, such as standardized EuroFlow approaches, have revealed distinct memory B-cell defects associated with specific clinical phenotypes in CVID (13, 14). Similarly, the EUROclass classification provides a framework for subclassifying CVID patients based on B-cell subset distributions derived from flow cytometric analysis, enabling a more refined stratification of immunological and clinical heterogeneity (15).

The predominant mucosal antibody is secretory IgA (sIgA), which exhibits superior antigen binding, agglutination, and neutralization capabilities compared to serum IgA (16). Mucosal antibodies may also have broader binding capacity than serum antibodies (17). Rather than being a peripheral extension of systemic immunity, mucosal immunity is organized and regulated largely independently. Mucosa-associated lymphoid tissues (MALT) harbor immune cell populations that are phenotypically and functionally different from those circulating in the systemic compartment (18). CVID patients present with decreased IgA levels and/or broader mucosal immune dysfunction, consistent with their increased susceptibility to respiratory and gastrointestinal infections, since most of the IgA in the body is present in the mucosal secretions (19). Growing evidence suggests that the gut microbiota serves as a potential contributor to both immune dysregulation and the inflammatory disease manifestations observed in CVID, also underscoring the importance of mucosal immune homeostasis (20, 21). Given that mucosal surfaces serve as the primary entry points for many pathogens to which CVID patients are particularly vulnerable, investigating mucosal immunity offers critical insights into the underlying disease mechanisms.

To gain deeper insights into the immunological background of CVID patients without a known genetic cause, we employed a genealogical research approach including both affected and non-affected first- and second-degree family members. Using blood samples from nine CVID index cases and their family members (n = 58), we performed high-dimensional flow cytometry to analyze the B-cell compartment alongside mucosal and serum antibody profiling. This integrative approach aimed to provide a more comprehensive understanding of immune dysregulation in CVID and to explore its potential heritability within affected families.

## Methods

### Study population

Patients were enrolled at the pediatric infectious diseases and immunology department of the Amalia Children’s Hospital, Radboudumc, Nijmegen, and at the department of internal medicine, Radboudumc, Nijmegen, after informed consent was provided by the subject and/or their legal representatives. The study was approved by the Medical Ethics Committee in Nijmegen, the Netherlands (Trial Registry ID: NL69477.091.19). Index patients were included if they fulfilled the ESID-defined CVID diagnosis according to the ESID registry (2014) (12), and if participation of at least four family members (siblings > 16 years of age, parents, or grandparents) was possible. All family members had to provide informed consent independently of the index case in order for the family to be eligible for inclusion. All index patients underwent genetic screening as part of their diagnostic workup. No pathogenic variants were identified through *in silico* analysis using the inborn errors of immunity (IEI) gene panel (latest update in 2021) (22). ESID-defined CVID patients with a known monogenetic cause of the disease were excluded from the study. Additional exclusion criteria were: infections (requiring antibiotic treatment) within three months prior to inclusion, surgeries within six months prior to the study or family members with significant disorders such as immunodeficiencies (excluding CVID), cancer, oral steroid therapy.

### Questionnaire

Family members were asked to complete a questionnaire regarding relevant medical history, focusing on signs or symptoms suggestive of primary immunodeficiency [adapted from (23)] (**Supplementary Table 1**).

### Sample collection

EDTA venous blood (9 ml) was drawn from the index cases and their family members.

In addition, nasal mucosal lining fluid (MLF) samples, a saliva sample, and a 0.3 ml fingerprick blood sample were collected from participants to compare antibody concentrations in serum and mucosal samples. MLF was collected using a Nasosorption™ FX·i device containing synthetic absorptive matrix (SAM) strips (Mucosal Diagnostics). SAM strips were gently inserted into the right nostril of the study subject and placed along the surface of the inferior turbinate. The index finger was lightly pressed on the nostril to keep the nasosorption device in place and to allow mucosal lining fluid absorption. After 60 seconds, the nasosorption device was placed back in the protective plastic tube and was first stored at -20°C and then transferred for long term storage to −80°C until further analyses. The MLF was eluted by pipetting 300 µL of elution buffer (PBS/1% BSA/0.05% Tween20/0.05% azide) into a 1.5 mL microcentrifuge tube containing a filter cup with a cellulose acetate membrane and placed on ice for 30 minutes to ensure that the filter membrane was blocked to prevent nonspecific protein binding. The SAM strips were detached from the holder using sterile forceps and placed into the buffer-containing filter in the microcentrifuge tube. Samples were then centrifuged for 20 minutes at 16,000 × *g* at 4 °C. After centrifugation, the filter cup containing the SAM strip was removed, and the eluate was stored at −80 °C until further analyses.

Saliva was collected using an Oracol Saliva Collection system. This device is designed to be used in a similar way to a toothbrush. Saliva is collected by rubbing the sponge swab firmly along the gum (at the base of the teeth if present), for about one minute, until the sponge is wet. Once the sponge was sufficiently wet, the device was replaced in the protective plastic tube and stored at −80 °C until further testing.

### Flow cytometry

All blood samples were processed and analyzed using EuroFlow Standard Operating Protocols (24). Within 12 hours after collection, blood was lysed using ammonium chloride and 10^7^ leukocytes were stained with a multicolor B-cell antibody panel targeting surface markers and intracellular immunoglobulin to specifically detect memory B-cell and plasma cell subsets, as previously described (14). In addition, 2.5x10^6^ cells were stained with a Primary Immunodeficiency Orientation Tube (PIDOT) to determine the general leukocyte subsets (25). For the B-cell tube, >5x10^6^ cells were acquired using either an Aurora (Cytek Bioscience) or a Lyric (BD Bioscience) flow cytometer. The PIDOT tube was measured using a Canto (BD Bioscience) or Lyric flow cytometer. Flow cytometry data were analyzed using Infinicyt software (Cytognos SL, Salamanca, Spain). An automated database analysis was used for analyzing the PIDOT tubes (25). The B-cell tube was analyzed as previously described by Blanco et al., and the results of all samples were compared to age-specific normal reference values (14, 26). Cell counts were calculated using the white blood cell count.

### Antibody profiling

For the measurement of total IgA and IgG levels in saliva, MLF and serum, we developed an in-house fluorescent-bead-based multiplex immunoassay. The targets, IgA and IgG, were bound to microspheres with distinct fluorescence excitation and emission spectra through carbodiimide coupling. 12.5x10^6^ beads (Magplex microspheres, Luminex^®^) were washed in MES activation buffer (50 mM MES (Sigma) in distilled water, pH 6.0) and activated by the addition of 5 mg/ml 1-ethyl-3-[3-dimethylaminopropyl]carbodiimide hydrochloride (EDC, ThermoFischer Scientific) and 5 mg/ml N-hydroxy-sulfosuccinimide (sulfo-NHS, ThermoFischer Scientific) and rotated for 20 minutes in the dark. After washing the activated beads, they were incubated with mouse anti-human IgA (BioLegend) or mouse anti-human IgG (BD Biosciences) for two hours in the dark and stored in high salt phosphate buffered saline (PBS (Lonza) + 0.8% NaCl + 0.02% KCl + 0.142% Na_2_HPO_4_, 0.024% KH_2_PO_4_) containing 0.1% bovine serum albumin (BSA), 0.02% Tween-20, and 0.05% sodium azide at 4 °C in the dark until use. For all measurements, samples, standards, and quality controls were diluted in assay buffer (high salt PBS + 1% BSA + 0.5% Tween-20). After obtaining informed consent, serum samples were collected from healthcare workers (without underlying conditions) and used as quality controls. Four quality control samples were included on every plate to correct for plate and batch variability. Saliva and MLF samples were diluted 1:10,000, while serum samples were diluted 1:100,000 for total IgA and IgG quantification. Samples with signals exceeding the upper limit of the standard curve were further diluted to fall within the measurable range. All measurements were conducted using the Luminex^®^ FlexMap3D System.

### Statistical analysis

Aberrancies in the lymphocyte compartment were expressed as fold changes relative to the 5^th^ and 95^th^ percentile of the age-specific normal reference values (25, 26).

SPSS (version 26, IBM, Armonk, NY) and RStudio (www.rstudio.com) were used for statistical analysis. Comparisons of characteristics between families with a positive versus negative family history for CVID were conducted with the Mann-Whitney U test for continuous variables and the χ^2^ test for categorical variables. Correlations between antibody levels and/or cell types were assessed using a Spearman’s rank correlation test. To measure the effect of family history and symptoms on antibody levels, a linear regression model was used. Family history and symptoms were taken as independent variables, and the antibody levels as dependent variables. In these regression analysis age was used as a covariate. A p value < 0.05 was considered significant. Correction for multiple testing was not applied due to the exploratory nature of the study.

## Results

### Characteristics and medical history of CVID patients and their family members

A total of 58 participants from nine families were included in the study, with five families (n = 32) having no family history of CVID (FH- families) and four families (n = 26) having a positive family history of CVID (FH+ families). The median age of index CVID patients was 11 years (range 4-22). Seven index patients were female and two were male. All index patients received IgG suppletion therapy. The clinical characteristics of the index patients are outlined in **Table 1**. The median age of family members was 56.5 years (range 6-84). The proportion of females to males, and age, was similar between groups. Except for the occurrence of immunodeficiencies (p < 0.01), there were no significant differences in characteristics between the two family groups. In the FH+ families, eight family members had a diagnosed immunodeficiency, further referred to as affected family members. Within these, CVID was the most frequently diagnosed immunodeficiency (n = 4), followed by IgG subclass deficiency (n = 3) and hypogammaglobulinemia (n = 1) (**Table 2**).

**Table 1:** Characteristics of index patients.

Characteristic	Index cases (n=9)
Age (y) *median (IQR)*	11 (9-17)
Gender *female n(%)*	7(77.8)
Therapy *n(%)*	
IVIG SCIG	2 (22.2) 7 (77.8)
Antibiotic prophylaxis *n(%)*	
Yes No	3 (33.3) 6 (66.7)
Hypogammaglobulinemia *n(%)*	
Yes No	7 (77.8) 2 (22.2)
Impaired vaccine response *n(%)*	
Yes No	8 (88.9) 1 (11.1)
IgA deficiency *n(%)*	
Yes No	6 (66.7) 3 (33.3)

IVIG, intravenous immunoglobulins, SCIG, subcutaneous immunoglobulins.

**Table 2:** Characteristics of the family members.

Characteristic	Family history negative (n=27)	Family history positive (n=21)
Age (y) *median (IQR)*	58.0 (46.5-73.5)	52.0 (36.0-67.0)
Gender *female n(%)*	14 (51.9)	11 (52.4)
Immunodeficiency *n(%)*		
Yes CVID Non-speccific Hypogammaglobulinemia IgG subclass deficiency	0 (0)	8 (38.1) 4 (50) 1 (12.5) 3 (37.5)

Index patients are excluded from this table.

To further explore potential subclinical immune dysfunction, all family members (both from the FH- and FH+ group) who did not meet the ESID criteria for immunodeficiency, further referred to as non-affected family members (n = 41), were asked to complete a questionnaire assessing medical history and symptoms suggestive of PID. Responses revealed that even family members without a diagnosed PID reported symptoms indicative of possible underlying immune dysfunction. Overall, 17 otherwise healthy family members answered ‘Yes’ to at least one item on the questionnaire (**Supplementary Table 2**). Despite differences in the prevalence of immunodeficiencies between FH+ and FH- families, the frequency of self-reported symptoms did not differ significantly. Notably, five out of these 41 non-affected family members reported more than eight upper respiratory tract infections (URTIs) per year (n = 3 out of the FH-, n = 2 out of the FH+).

### Aberrant B- and T-cell profiles in CVID patients

**Figure 1:**
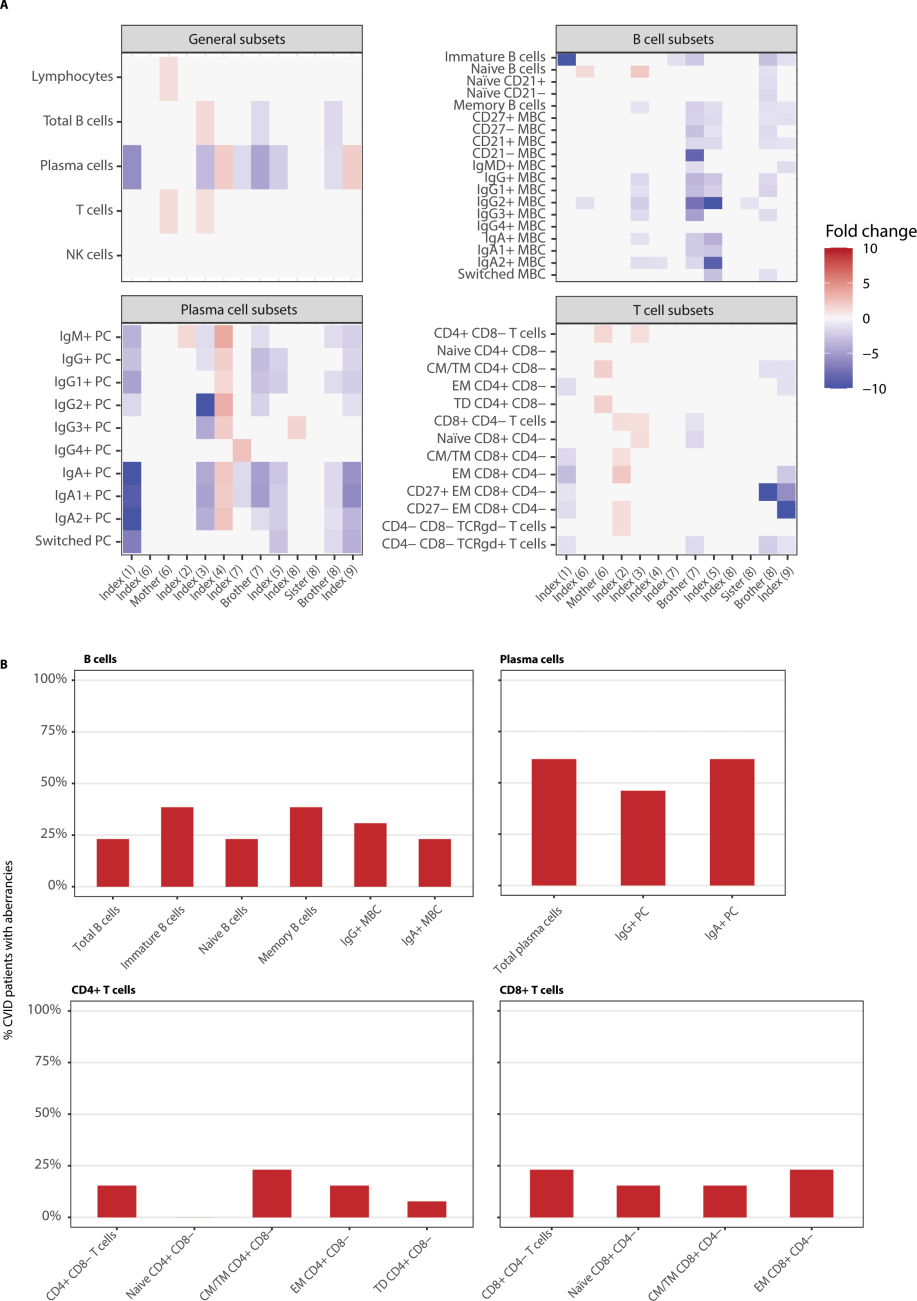
Immunophenotyping of CVID patients presents heterogeneous abnormalities in their B- and T-cell compartment. **(A)** Absolute counts were compared to age-specific normal reference values and expressed as a foldchange relative to the 5^th^ and 95^th^ percentile of those reference values and presented in a heatmap. X axis shows the index patient or affected family members and to which family they belong. NK, natural killer; PC, plasma cell; MBC, memory B-cell; CM, central memory; TM, transitional memory; EM, effector memory; TD, terminally differentiated. **(B)** shows the frequencies of aberrancies in the major subsets for the CVID patients. Aberrancies were defined as an absolute cell count for the relevant population that was below the 5^th^ or above the 95^th^ percentile of age-specific normal reference values.

Immunophenotyping was performed on blood from 56 participants (i.e. index patients and family members) to determine the distribution of 46 leukocyte subsets, including 31 B-cell and 15 T-cell subsets. Two measurements could not be included in this analysis due to missing data. The cohort included 13 CVID patients (index patients and affected family members with CVID), all of whom were receiving immunoglobulin replacement therapy at the time of analysis. A significant reduction in total plasma cells of 1.4- to 5.9-fold was observed in about half of the CVID patients (**Figures 1A, B**, **Table 3**). Reduced IgA+ (1.5- to 10.5-fold decrease) and IgG+ (1.3- to 3.2-fold decrease) plasma cells were observed in 64% of the patients, while CD27^+^ memory B-cells were decreased in one third of the CVID patients (1.1- to 2-fold decrease), indicating impaired B-cell maturation. In adult CVID patients, the EUROclass classification can provide different subgroups that correlate with clinical manifestations (15). The immune profiles of the CVID patients were compared to the EUROclass subgroups. All patients fit into the SmB+CD21norm subgroup. Immune aberrancies in CVID patients were not limited to the B-cell compartment, but were also observed in the T-cell compartment. Seven out of 13 CVID patients exhibited abnormalities in both the CD4^+^ and the CD8^+^ T-cells, which consisted mostly of lowered memory subset numbers, indicating a broader immunological dysfunction (**Figures 1A, B**).

**Table 3:** Frequency of aberrancies in B- and T-cell subpopulations in CVID patients.

Cell population	CVID patients, n=1 3 [n(%)]
Lymphocytes	1(8)
Total B cells	3(23)
Immature B cells	5(38)
Total Naive B cells	3(23)
Naïve CD21+	1(8)
Naïve CD21-	1(8)
Total Memory B cells	5(38)
CD27+ MBC	4(31)
CD27- MBC	3(23)
CD21+ MBC	4(31)
CD21- MBC	1(8)
IgMD+ MBC	2(15)
Total IgG+ MBC	4(31)
IgG1+ MBC	4(31)
IgG2+ MBC	5(38)
IgG3+ MBC	3(23)
IgG4+ MBC	0(0)
Total IgA+ MBC	3(23)
IgA1+ MBC	2(15)
IgA2+ MBC	4(31)
Total Switched MBC	2(15)
Total Plasma cells	8(62)
IgM+ PC	7(54)
Total IgG+ PC	6(46)
IgG1+ PC	6(46)
IgG2+ PC	5(38)
IgG3+ PC	3(23)
IgG4+ PC	1(8)
Total IgA+ PC	8(62)
IgA1+ PC	8(62)
IgA2+ PC	7(54)
Total Switched PC	4(31)
Total T cells	2(15)
Total CD4+ CD8- T cells	2(15)
Naive CD4+ CD8-	0(0)
CM/TM CD4+ CD8-	3(23)
EM CD4+ CD8-	2(15)
TD CD4+ CD8-	1(8)
Total CD8+ CD4- T cells	3(23)
Naïve CD8+ CD4-	2(15)
CM/TM CD8+ CD4-	2(15)
EM CD8+ CD4-	3(23)
CD27+ EM CD8+ CD4-	3(23)
CD27- EM CD8+ CD4-	3(23)
CD4- CD8- TCRgd- T cells	1(8)
CD4- CD8- TCRgd+ T cells	4(31)
NK cells	0(0)

Data shows the number of CVID patients of whom an aberrancy in the relevant subpopulation was detected and the percentage.

### Family members of CVID patients show aberrant immune profiles

**Figure 2:**
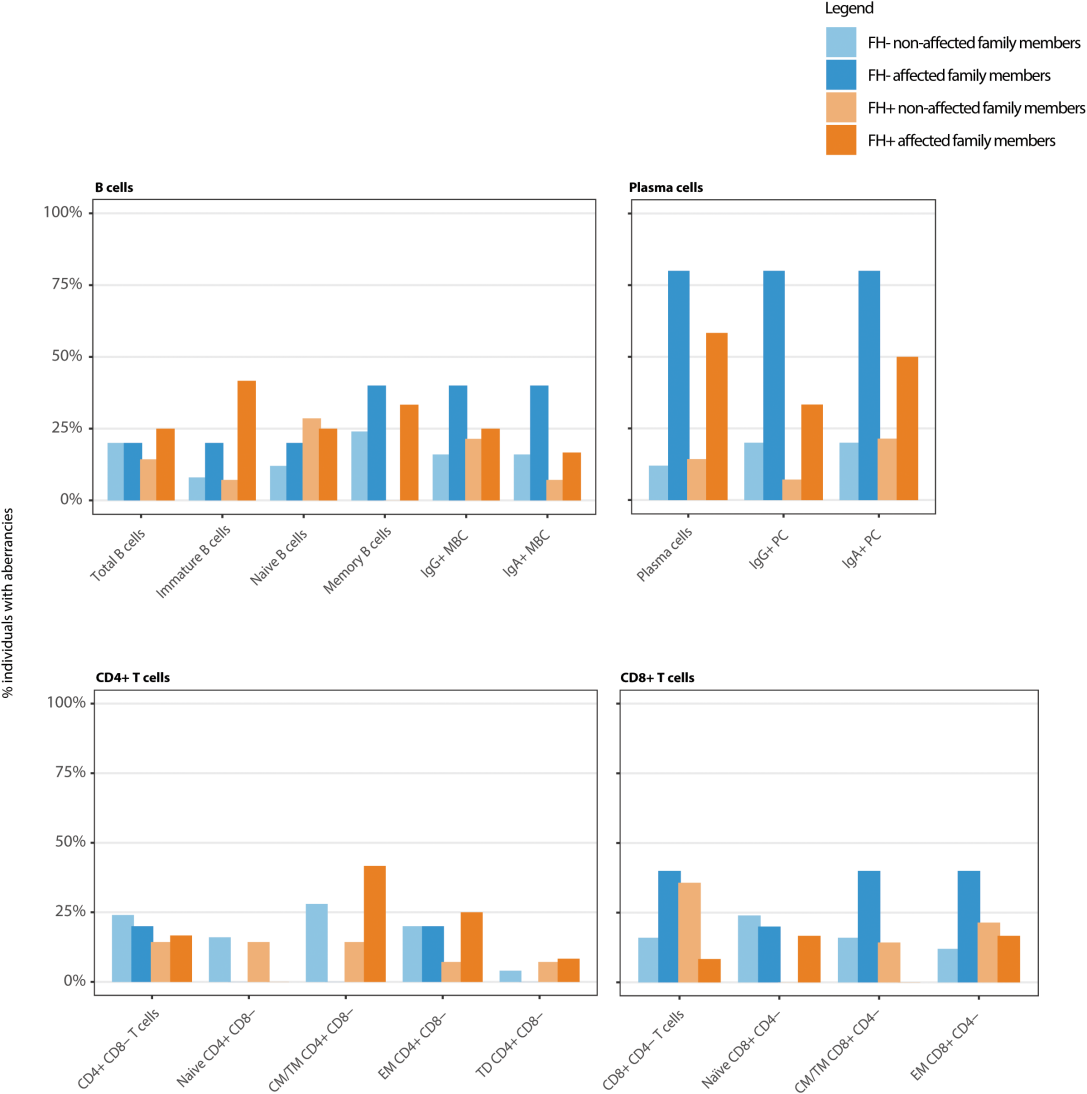
Immunophenotyping of index and family members shows aberrancies in the B- and T-cell compartment in both affected and non-affected family members in both family history negative (FH-) as family history positive (FH+) families. Bars show the frequencies of aberrancies in the major subsets per affected and non-affected family members in FH- and FH+ families. Aberrancies were defined as an absolute cell count for the relevant population that was below the 5^th^ or above the 95^th^ percentile of age-specific normal reference values.

**Figure 3:**
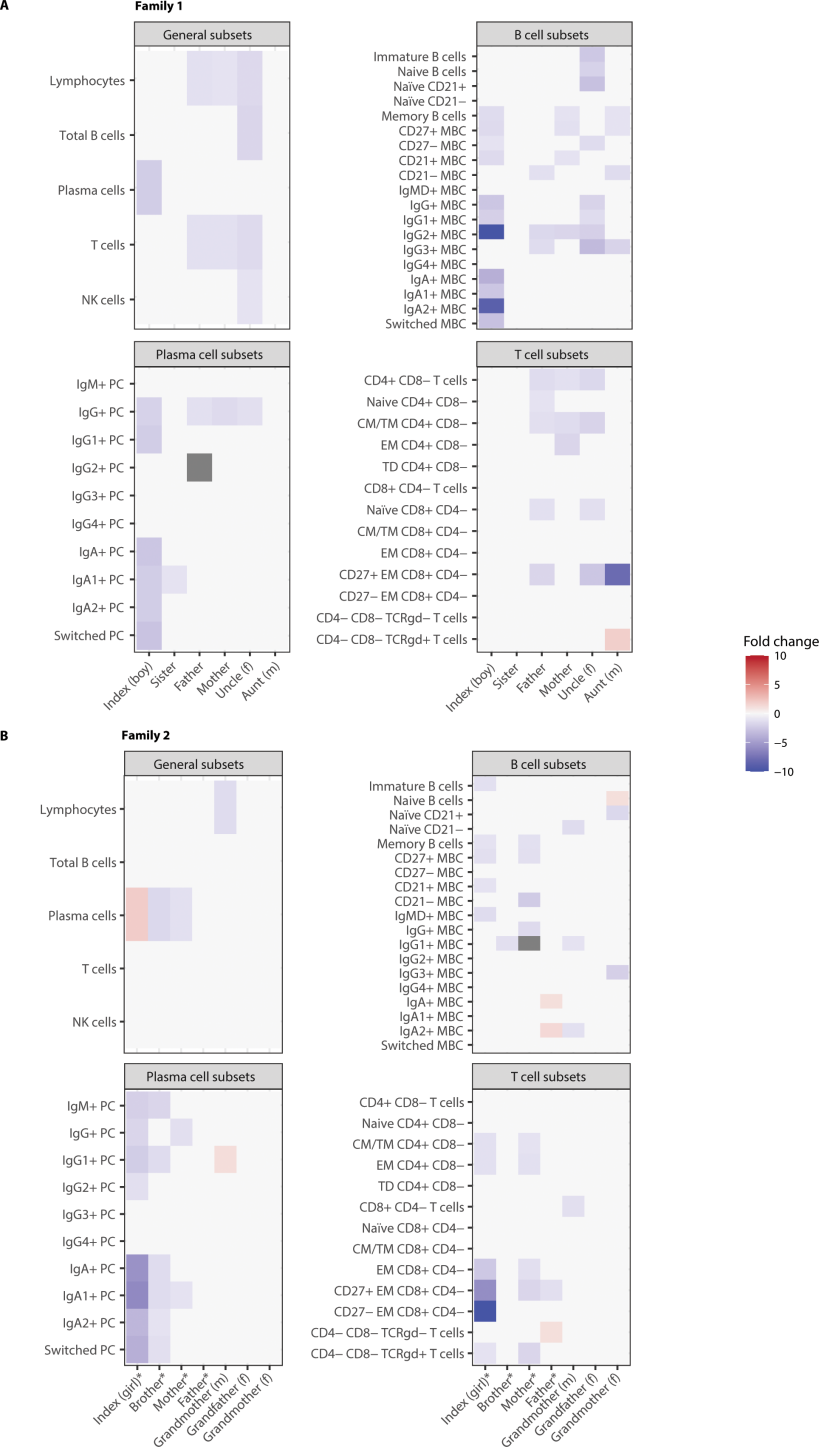
Immunophenotyping of index and family members shows similar patterns of aberrancies in the B- and T-cell compartment in a family history negative (FH-) and family history positive (FH+) family. Absolute counts were compared to age-specific normal reference values and expressed as a foldchange relative to the 5th and 95th percentile of those reference values. **(A)** Family 1 is the family with a negative family history. **(B)** Family 2 is the family with a positive family history. (m) indicates a relative from mothers side, (f) indicates a relative from fathers side. NK, natural killer; PC, plasma cell; MBC, memory B-cell; CM, central memory; TM, transitional memory; EM, effector memory; TD, terminally differentiated. * indicates the affected family members. Grey fields indicate that there were no measurable cells present of this cell type.

Interestingly, abnormalities in the immunophenotyping were also detected in family members, irrespective of a formal ESID diagnosis. Decreases in memory B-cell subsets were observed across all families (FH+ and FH-) and both in affected and non-affected family members (**Figures 2**, **3**; **Supplementary Figures 1**, **2**; **Table 4**). In five families, at least one member had reduced CD27+ memory B-cells. Of these five families, reduced CD27+ memory B-cells were observed in affected and non-affected family members. Reduction in plasma cell subsets was found in eight out of the nine families, and in seven out of those eight families the reductions also occurred in non-affected family members. An IgA+ plasma cell reduction was found in eight out of nine families. Lowered serum IgA levels were not consistently observed in family members with reduced IgA+ plasma cells (**Supplementary Tables 3**-**9**). This reduction in IgA+ plasma cells was also found in non-affected members from six families (n = 3 FH-, n = 3 FH+). Apart from aberrancies in the B-cell compartment, alterations in the T-cell compartment were found. A reduction in various CD4+ and CD8+ T-cell subsets was found in all families, both in affected and non-affected family members (**Figure 2**). In addition, the combination of T-cell and B-cell reductions was observed in at least one member of all the families, and in eight out of the nine families it was found in a non-affected member. To conclude, looking at patterns within families, different members showed aberrancies in the same subsets. In eight out of nine families, this was also seen in the so called non-affected family members, without formal ESID diagnosis.

**Table 4:** Frequencies of aberrancies in B- and T-cell subpopulations in affected and non-affected family members in family history negative and family history positive families.

	Family history negative		Family history positive	
	Non-affected, n=25 (n(%))	Affected, n=5 (n(%))	Non-affected, n=14 (n(%))	Affected, n=12 (n(%))
Lymphocytes	8(32)	0(0)	5(36)	2(17)
Total B cells	5(20)	1(20)	2(14)	3(25)
Immature B cells	2(8)	1(20)	1(7)	5(42)
Total Naive B cells	3(12)	1(20)	4(29)	3(25)
Naïve CD21+	1(4)	0(0)	2(14)	2(17)
Naïve CD21-	3(12)	0(0)	2(14)	1(8)
Total Memory B cells	6(24)	2(40)	0(0)	4(33)
CD27+ MBC	4(16)	1(20)	0(0)	4(33)
CD27- MBC	3(12)	1(20)	1(7)	2(17)
CD21+ MBC	5(20)	1(20)	0(0)	3(25)
CD21- MBC	8(32)	0(0)	1(7)	2(17)
IgMD+ MBC	5(20)	0(0)	2(14)	2(17)
Total IgG+ MBC	4(16)	2(40)	3(21)	3(25)
IgG1+ MBC	2(8)	2(40)	2(14)	3(25)
IgG2+ MBC	6(24)	2(40)	4(29)	3(25)
IgG3+ MBC	8(32)	1(20)	5(36)	3(25)
IgG4+ MBC	0(0)	0(0)	2(14)	0(0)
Total IgA+ MBC	4(16)	2(40)	1(7)	2(17)
IgA1+ MBC	4(16)	1(20)	2(14)	1(8)
IgA2+ MBC	6(24)	3(60)	2(14)	2(17)
Total Switched MBC	2(8)	1(20)	1(7)	1(8)
Total Plasma cells	3(12)	4(80)	2(14)	7(58)
IgM+ PC	3(12)	4(80)	0(0)	4(33)
Total IgG+ PC	5(20)	4(80)	1(7)	4(33)
IgG1+ PC	1(4)	3(60)	1(7)	4(33)
IgG2+ PC	3(12)	3(60)	1(7)	2(17)
IgG3+ PC	0(0)	2(40)	0(0)	1(8)
IgG4+ PC	1(4)	0(0)	0(0)	1(8)
Total IgA+ PC	5(20)	4(80)	3(21)	6(50)
IgA1+ PC	5(20)	4(80)	3(21)	7(58)
IgA2+ PC	5(20)	4(80)	1(7)	4(33)
Total Switched PC	4(16)	2(40)	2(14)	4(33)
Total T cells	5(20)	1(20)	3(21)	2(17)
Total CD4+ CD8- T cells	6(24)	1(20)	2(14)	2(17)
Naive CD4+ CD8-	4(16)	0(0)	2(14)	0(0)
CM/TM CD4+ CD8-	7(28)	0(0)	2(14)	5(42)
EM CD4+ CD8-	5(20)	1(20)	1(7)	3(25)
TD CD4+ CD8-	1(4)	0(0)	1(7)	1(8)
Total CD8+ CD4- T cells	4(16)	2(40)	5(36)	1(8)
Naïve CD8+ CD4-	6(24)	1(20)	0(0)	2(17)
CM/TM CD8+ CD4-	4(16)	2(40)	2(14)	0(0)
EM CD8+ CD4-	3(12)	2(40)	3(21)	2(17)
CD27+ EM CD8+ CD4-	5(20)	1(20)	2(14)	4(33)
CD27- EM CD8+ CD4-	4(16)	2(40)	1(7)	1(8)
CD4- CD8- TCRgd- T cells	6(24)	1(20)	0(0)	1(8)
CD4- CD8- TCRgd+ T cells	6(24)	1(20)	3(21)	4(33)
NK cells	4(16)	0(0)	3(21)	0(0)

Data shows the number of people of whom an aberrancy in the relevant subpopulation was detected and the percentage in the subgroup.

### Exploring patterns within FH- and FH+ families

As family members of CVID patients show aberrant immune profiles, patterns within FH− and FH+ families were explored. **Figure 3** illustrates patterns within two families, one FH- family (**Figure 3A**) and one FH+ family (**Figure 3B**), with corresponding characteristics shown in **Tables 5**, **6**. These two families were highlighted for having non-affected family members with aberrancies in their lymphocyte compartment. In several members of both the FH− and FH+ families, B cell subsets were reduced up to a 5- to 10-fold decrease compared with the 5^th^ percentile of the reference values.

**Table 5:** Characteristics and antibody levels in serum, saliva and mucosal lining fluid (MLF) of a representative family history negative (FH-) family and family history positive(FH+) family.

Family 1 (FH-)	Index (boy)	Sister	Father	Mother	Uncle (f)	Aunt (m)
Characteristics						
Age (years)	18	20	54	50	58	53
Diagnosis	CVID	–	–	–	–	–
Therapy	SCIG	–	–	–	–	–
Questionnaire	–	Recurrent infections	–	–	–	>8 URTIs/year
Antibody levels (ng/ml)						
Serum IgG	9.90E+06	7.81E+06	6.93E+06	7.49E+06	8.09E+06	Missing^+^
Serum IgA	4.26E+05	1.32E+06	2.54E+06	8.27E+05	2.13E+06	Missing^+^
Saliva IgG	Missing	8.82E+04	7.70E+04	2.61E+05	Missing^	Missing^+^
Saliva IgA	Missing	4.85E+06	4.99E+06	9.73E+06	Missing^	Missing^+^
MLF IgG	1.54E+05	4.18E+04	4.32E+04	9.77E+04	1.53E+04	Missing^+^
MLF IgA	4.36E+05	4.82E+05	1.25E+06	2.26E+05	1.70E+05	Missing^+^

URTI, upper respiratory tract infection. All serum antibodies were measured in the serum collected from the extra finger prick blood during the study. ^ insufficient material for analysis, ^+^ no sample received.

**Table 6:** Characteristics and antibody levels in serum, saliva and mucosal lining fluid (MLF) of a representative family history negative (FH-) family and family history positive(FH+) family.

Family 2 (FH+)	Index (girl)	Brother	Father	Mother	Grand-father (f)	Grand-mother (f)	Grand-mother (m)
Characteristics							
Age (years)	17	19	52	49	81	84	73
Diagnosis	CVID	Hypogammaglobulinemia	IgG1 and IgG3 subclass deficiency; IgM deficiency	Hypogammaglobulinemia; IgG1 subclass deficiency	–	–	–
Therapy	SCIG	–	–	–	–	–	–
Questionnaire	–	–	–	–	–	DM	Thyroid disease; DM
Antibody levels (ng/ml)							
Serum IgG	6.15E+06	Missing^	4.27E+06	3.88E+06	8.52E+06	1.19E+07	1.01E+07
Serum IgA	3.21E+05	Missing^	1.00E+06	1.81E+06	4.23E+06	3.12E+06	3.67E+06
Saliva IgG	6.32E+04	Missing^	2.38E+04	3.67E+04	Missing^	Missing^	Missing^
Saliva IgA	8.88E+05	Missing^	6.09E+05	1.64E+06	Missing^	Missing^	Missing^
MLF IgG	1.62E+04	1.90E+04	2.86E+04	4.57E+03	7.57E+03	5.73E+04	8.38E+03
MLF IgA	4.60E+05	1.93E+05	2.04E+05	1.63E+05	1.67E+05	1.07E+05	8.65E+05

URTI, upper respiratory tract infection. All serum antibodies were measured in the serum collected from the extra finger prick blood during the study. ^ insufficient material for analysis, ^+^ no sample received.

In the FH- family (family 1) shown in **Figure 3A**, the index patient, mother, and maternal aunt showed reduced CD27+ memory B-cells, indicating a possible shared immunological trait on the mother’s side. The index patient, father, and paternal uncle all exhibited reduced IgG2^+^ memory B-cells, suggesting a shared immunological trait on the father’s side. However, since the mother also displayed the same abnormality, this pattern does not appear to follow a strict genealogical inheritance. Notably, the index patient exhibited a more pronounced reduction in IgG2^+^ memory B-cells, which may be due to the fact that this trait was present in both parental lineages, potentially leading to an additive effect on immune dysfunction. The index patient also exhibited a reduction in plasma cells, including both IgA+ and IgG+ plasma cells. Serum IgG and IgA levels appeared to be normal in all family members. The index patient had the lowest serum IgA level, but high serum IgG levels, which is in line with the index case receiving IgG suppletion therapy that does not contain IgA (**Table 3**). Furthermore, while the index patient exhibited no abnormalities in the T-cell compartment, both the father and the paternal uncle showed reduced CD4^+^ and CD8^+^ T-cell subset counts, suggesting an altered T-cell compartment in these two family members despite the absence of a diagnosed immunodeficiency.

In the FH+ family (family 2) shown in **Figure 3B**, the sibling was diagnosed with hypogammaglobulinemia, the father with an IgG1, IgG3, and IgM deficiency, and the mother with hypogammaglobulinemia and an IgG1 subclass deficiency (**Table 4**). Both the index patient and the sibling showed a similar pattern of plasma cell subset depletion, with markedly reduced IgA^+^ plasma cells, suggesting a shared immune dysfunction, possibly in the mucosal compartment, with the index patient showing low saliva IgA levels and the sibling showed lowered MLF IgA levels. Beyond the plasma cell compartment, the index patient and mother displayed a significant reduction in CD27^+^ memory B-cells, indicating impaired B-cell maturation. Additionally, both the index patient and the mother shared similar T-cell compartment aberrancies, further emphasizing a recurring immune phenotype within this family. In contrast, the father did not show major lymphocyte count abnormalities, despite being diagnosed with a PID. Serum analysis revealed lowered IgG and IgA levels in the index patient and lowered IgG levels in both the parents. Unfortunately, no serum sample was available from the sibling, preventing a serum IgA assessment (**Table 4**).

### Discordance of mucosal and systemic IgA and IgG levels

Significantly reduced IgA+ plasma cells were observed in both CVID patients and some non-affected family members (n = 8) (**Figure 1**, **Figure 3**; **Supplementary Figures 1**, **2**; **Tables 3**, **4**). To further investigate mucosal immunity in CVID, IgA and IgG concentrations were measured in MLF (n = 54), saliva (n = 32) and serum (n = 52). Four individuals were excluded due to death (n = 1), ongoing cancer treatment (n = 1), or loss of contact (n = 2). In addition, IgA and IgG levels could not be assessed in 26 subjects because of insufficient saliva samples (**Supplementary Tables 2**-**8**). Correlations between mucosal and serum IgA and IgG were evaluated in both CVID patients and non-affected family members to determine whether serum IgA and IgG levels reflect mucosal levels.

**Figure 4:**
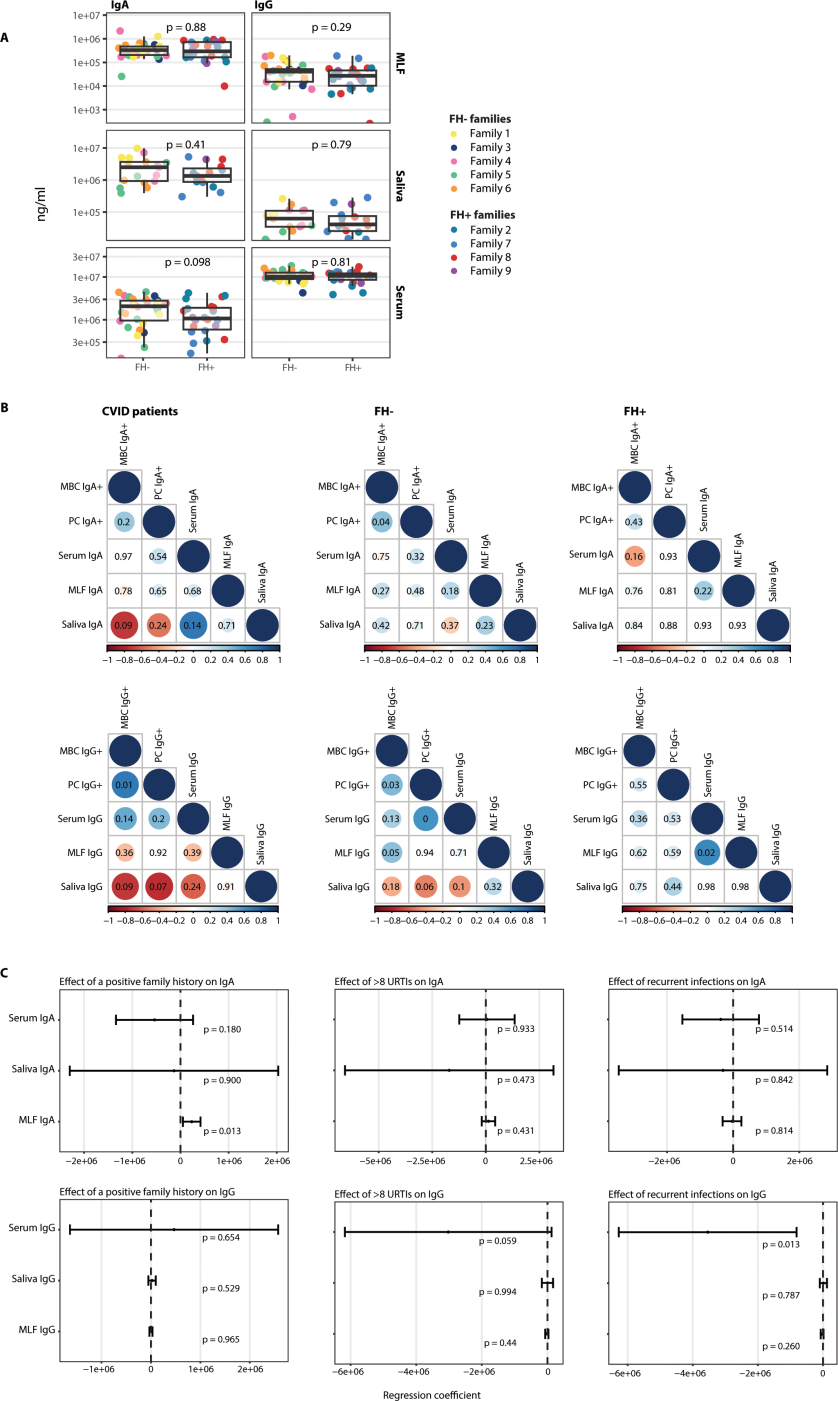
Systemic and mucosal IgG and IgA do not differ between family members from the family history positive families (FH+) and family history negative families (FH-). The correlation mucosal and systemic Ig levels and plasma cells (PC) and memory B-cells (MBC) and the association between a positive family history, >8 upper respiratory tract infections (URTIs) and recurrent (bacterial) infections on mucosal and systemic Ig levels. For the index patients samples were taken during Immunoglobulin replacement therapy. **(A)** The IgA and IgG levels (ng/ml) in serum, mucosal lining fluid (MLF) and saliva for FH- vs FH+ families. Colors indicate the different families. P value was determined using a Mann-Whitney U test. **(B)** The correlations of systemic and mucosal Igs and memory B-cells (MBC) and plasma cells (PC) for the CVID patients (n=13), the non-affected members of the families with a negative family history for CVID (n=25) and the non-affected members of the families with a positive family history for CVID (n=14). All correlations are spearman correlation. Value within the circle shows the p value. **(C)** Forrest plots of the effect of a positive family history, >8 URTIs and recurrent (bacterial) infections on mucosal and systemic IgA and IgG using a linear regression, corrected for age.

Serum and mucosal IgG and IgA levels in FH− families were variable but did not differ significantly from those in FH+ families (**Figure 4A**). There were no significant correlations in systemic vs mucosal IgA levels in non-affected family members from either FH- or FH+ families (**Figure 4B**; **Supplementary Figure 3B**, **Supplementary Tables 9**, **10**), indicating that systemic IgA levels do not reflect mucosal IgA levels. For FH+ family members, there was a significant positive correlation between IgG levels in serum and MLF (**Figure 4B**; **Supplementary Figure 4B**, **Supplementary Tables 11**, **12**). In CVID patients, there were no significant correlations between systemic and mucosal IgA and IgG, and no significant correlation between immunoglobulin levels in MLF and saliva, further indicating that the mucosal and systemic immune compartments function independently in CVID patients, and treatment with systemic Ig suppletion does not affect the mucosal levels (**Figure 4B**; **Supplementary Figures 3A**, **4A**, **Supplementary Tables 13**, **14**).

In the non-affected family members, the effect of having a positive family history, having > 8 URTIs per year, or having recurrent (bacterial) infections, was tested using linear regression corrected for age. The non-affected family members of families with a FH+ had higher MLF IgA levels than those with a FH-. There was no effect on serum IgA, saliva IgA, serum IgG, saliva IgG, and MLF IgG. Five non-affected family members reported > 8 URTIs per year. There were no significant differences in serum IgA, MLF IgA, or saliva IgA between those with frequent URTIs and those without. Serum IgG showed a trend towards being lower in non-affected family members with frequent URTIs. MLF IgG and saliva IgG did not differ between those with or without frequent URTIs. Additionally, seven out of 42 non-affected family members reported recurrent infections. Serum IgG was lower in non-affected family members that reported recurrent infections. Serum IgA, MLF IgA, saliva IgA, MLF IgG, and saliva IgG did not differ between those with recurrent infections or without (**Figure 4C**; **Supplementary Table 15**).

### Patterns of immune cell distribution and antibody levels

We wanted to assess whether the aberrancies found in the immune cell compartments correlated with systemic and mucosal antibody levels. For the CVID patients, no significant correlations were found between memory B-cells, plasma cells, and systemic and mucosal antibody levels in IgA. Saliva IgA did show a trend toward negative correlation with IgA+ memory B-cells (**Figure 4B**, **Supplementary Figure 3A**, **Supplementary Table 13**). Similar observations were made for saliva IgG and IgG+ memory B-cells, and saliva IgG and IgG+ plasma cells (**Figure 4B**, **Supplementary Figure 4A**, **Supplementary Table 14**). This same trend of negative correlation was found in the non-affected family members of FH- families for saliva IgG and IgG+ plasma cells (**Figure 4B**, **Supplementary Figure 4B**, **Supplementary Table 11**). Regarding IgG+ cells in the CVID patients, there was a significant correlation between memory B-cells and plasma cells. This correlation was also observed in FH- family members. For the family members of FH- families, there was a significant positive correlation between IgA+ memory B cells and plasma cells (**Figure 4B**, **Supplementary Figure 3B**, **Supplementary Table 9**). For FH- family members, there was a positive correlation between IgG+ plasma cells and serum IgG levels.

## Discussion

This study provides new insights into the immunological landscape of CVID and its familial patterns. We aimed to provide a more comprehensive understanding of immune dysregulation in CVID and its potential heritability within affected families. The clinical presentation of CVID is diverse, and patients can also have mild symptoms (27, 28). It is important to identify patients even if they only display mild symptoms, so they can be monitored for developing complications, such as bronchiectasis. Early identification allows for timely treatment, which may prevent the progression to serious complications (28–30).

In our study, we observed that immune deficiencies within FH+ families follow a complex pattern, with some immune traits shared among family members, but differing in severity. Interestingly, non-affected family members reported clinical symptoms that were similar between FH+ and FH− families. These symptoms were likely mild enough not to warrant medical attention, yet their immune profiles still showed alterations. This suggests that self-reported symptoms alone may not reliably differentiate individuals with a family history of CVID from those without, reinforcing the importance of objective immunophenotyping in family assessments.

Immunophenotyping of CVID patients revealed diverse aberrancies in both B and T-cell compartments. Whereas Blanco et al. (14) reported an almost complete absence of switched plasma cells in their CVID cohort, only seven out of 13 patients in our study showed a significant reduction in this subset. A recent study by Torres-Valle et al. (31), performing immunophenotyping on a large cohort of CVID patients (n = 209) has updated the different profiles based on plasma cell and memory B cell defects of CVID patients. They included a profile of patients with normal to reduced switched plasma cells and memory B cells, which fits with our CVID patients. Comparable results of normal to reduced switched memory B cells were observed in our study, with reductions identified in 3 out of 13 CVID patients. Interestingly, this reduction was also observed in some non-affected family members, suggesting that certain immunological defects may precede clinical disease onset or manifest along a spectrum of clinical severity, including asymptomatic courses.

The finding of T-cell aberrancies in our CVID patients is in line with previous studies. CVID patients have previously been described to have, amongst others, a decrease in regulatory T-cells and/or T follicular helper cells, and an expansion of CD8+ T-cells [reviewed in (32, 33)]. Due to limited total amount of blood, we were only able to investigate the T-cell compartment at a global level. For future studies, it would be of interest to assess the T-cell compartment in more detail and to investigate whether these known T-cell abnormalities are also present in the family members.

Previous studies have investigated serum antibody levels in family members of CVID patients, but did not perform in-depth analyses of both affected and non-affected first- and second-degree family members (34–37). In our cohort, we observed mild immune dysfunction in family members without a formal PID diagnosis, as evidenced by self-reported PID-like symptoms and flow cytometric abnormalities in some clinically healthy relatives. However, these self-reported symptoms did not consistently correlate with objective B-cell immunophenotyping results, highlighting the complexity of immune regulation within CVID families. Together, these findings indicate that clinical history alone is insufficient for assessing immune dysfunction, and that high-dimensional immune profiling is essential for detecting subclinical immune deviations. This approach may improve diagnostic accuracy, and guide timely and adequate treatment, which is essential to prevent irreversible damage caused by chronic, low-grade inflammation.

Current diagnostic tools for CVID primarily rely on blood-based analysis, including antibody levels and switched memory B-cell counts (12). While these alterations reflect germinal center disturbances, they remain indirect measures of germinal center function. Direct analysis of lymph nodes would be highly informative, but is hardly feasible. In rare cases where lymph nodes from CVID patients with breast cancer or lymphoma have been examined, clear germinal center abnormalities were observed (38–40). Clinical manifestations of CVID also suggest involvement of the mucosa. The gut mucosal immune system has previously been analyzed in CVID patients. In contrast, the mucosa of the upper respiratory tract is easily accessible, but has been minimally investigated in CVID patients. Our analysis of mucosal immunity revealed that systemic IgA deficiency in CVID patients does not correlate with reduced mucosal IgA levels. This finding suggests that systemic and mucosal immune compartments function independently, complicating the interpretation of systemic IgA levels with respect to mucosal immunity. Additionally, no significant differences in mucosal IgA or IgG levels were found between affected and non-affected participants, indicating that mucosal immunity in CVID may be more heterogeneous than previously thought (21, 41). Given the critical importance of IgA in mucosal defense, our findings raise important questions about whether mucosal-targeted therapeutic strategies, beyond conventional intravenous IgG (IVIG) therapy, could improve infection control in CVID patients (42).

Some limitations of our study should be considered. Due to the exploratory nature of the study, the number of index patients and family members included may have limited the statistical power. Additionally, this limits the ability to compare our cohort to relevant CVID classification systems like EUROclass (15). At the same time, although CVID can occur in children (often considered if > 4 years old), the EUROclass criteria were mainly developed based on adult values and may not be directly applicable to children due to immune system maturation.

Secondly, this analysis shows a snapshot of the immune system at a single timepoint and does not measure temporal changes. Longitudinal sampling could assess these changes. Another limitation is that the genetic screening of index patients does not include the most recently identified CVID-associated genes (i.e. panel updated in 2021). In large cohorts of up to 1300 inborn errors of immunity, the benefit of systematic reanalysis of exome data has shown a 1,7 percentage increase in conclusive molecular diagnosis (22). As such, we cannot completely exclude the presence of a variant in a novel CVID-associated gene, compared to what would have been detected if the study subjects had been included at present time. Furthermore, it is important to highlight that we used a screening panel for IEI genes and did not perform in-depth whole exome or genome sequencing, which could identify novel CVID-associated genes.

Finally, it should be noted that some family members in this study may potentially have an undiagnosed immunodeficiency. Although we can exclude CVID based on their immunoglobulin levels and clinical presentation, we cannot rule out the presence of a more subtle antibody defect, such as Specific Antibody Deficiency (SPAD), due to the fact that vaccine responses were not assessed for family members.

Collectively, our findings underscore the high degree of heterogeneity in CVID, both in familial patterns and immunophenotypic manifestations. The detection of immune aberrancies in asymptomatic family members suggests that certain immune traits may represent early indicators of immune dysfunction, although their predictive value for CVID development remains uncertain. Future studies should prioritize longitudinal immune profiling of at-risk relatives to determine whether early immune deviations correlate with disease progression or clinical outcomes.

## Data Availability

The raw data supporting the conclusions of this article will be made available by the authors, without undue reservation.

## Supplementary material

Supplementary Figure 1 Immunophenotyping of families with a negative history for CVID show aberrancies in the B- and T-cell compartment in both the index cases as non-affected family members. Absolute counts were compared to age-specific normal reference values and expressed as a foldchange relative to the 5^th^ and 95^th^ percentile of those reference values. (m) indicates a relative from mothers side, (f) indicates a relative from fathers side. NK, natural killer; PC, plasma cell, MBC, memory B-cell, CM, central memory; TM, transitional memory; EM, effector memory; TD, terminally differentiated. Grey fields indicate that there were no measurable cells present of this cell type.

Supplementary Figure 2 Immunophenotyping of families with a positive history for CVID shows aberrancies in the B- and T-cell compartment in both affected and non-affected family members. Absolute counts were compared to age-specific normal reference values and expressed as a foldchange relative to the 5^th^ and 95^th^ percentile of those reference values. (m) indicates a relative from mothers side, (f) indicates a relative from fathers side. NK, natural killer; PC, plasma cell; MBC, memory B-cell; CM, central memory; TM, transitional memory; EM, effector memory; TD, terminally differentiated. * indicates the affected family members. Grey fields indicate that there were no measurable cells present of this cell type.

Supplementary Figure 3 IgA correlations in CVID patients and their family members. Data shows the Spearman correlation coefficient. * p value ≤ 0.05, ** p value ≤ 0.01. **(A)** shows the IgA correlations of the CVID patients. **(B)** shows the correlations of the non-affected family members from family history negative families (red) and family history positive families (blue).

Supplementary Figure 4 IgG correlations in CVID patients and their family members. Data shows the Spearman correlation coefficient. * p value ≤ 0.05, ** p value ≤ 0.01, *** p value ≤ 0.001. **(A)** shows the IgG correlations of the CVID patients. **(B)** shows the correlations of the non-affected family members from family history negative families (red) and family history positive families (blue).
