# Supplementary material for: Familial patterns of immune dysregulation in CVID: insights from B- and T-cell phenotyping and antibody profiling
Source: Front Immunol. 2026 Mar 13;17:1741900. doi: 10.3389/fimmu.2026.1741900 (PMC13021423; doi:10.3389/fimmu.2026.1741900)
Supplement: Supplementary file 1 [file Table1.docx]

**Supplementary tables**

**Supplementary table 1. Questionnaire about relevant primary immunodeficiency (PID) history**

| **Do you have one or more of the following signs which are associated with an immunodeficiency** | |
| --- | --- |
| Recurrent (probable) bacterial infections? | YES/NO |
| > 8 upper respiratory tract infections per year? | YES/NO |
| >2 pneumonias per year? | YES/NO |
| >1 serious invasive infection like meningitis, osteomyelitis (bone infection), pneumonia, sepsis? | YES/NO |
| Infections with atypical presentation, who are unusually serious, or do not respond to treatment? | YES/NO |
| Using antibiotics for >2 months with (almost) no response? | YES/NO |
| Need of IV antibiotics to treat an infection? | YES/NO |
| Abscesses of internal organs? | YES/NO |
| Repeated abscesses of the skin? | YES/NO |
| Frequent yeast infections in mouth or mucous membranes that are hard to cure or recur quickly after stopping treatment? | YES/NO |
| Chronical or recurring episodes of diarrhea? | YES/NO |
| Poor weight evolution, unexplained weight loss or delayed growth? | YES/NO |
| Family members with similar symptoms? | YES/NO |
| Autoimmunity like thyroid disease, diabetes, autoimmune cytopenia, low blood cell counts, bowel diseases? | YES/NO |
| Complications after a vaccination? | YES/NO |
| Did a doctor ever found granulomas? | YES/NO |
| Recurrent unexplained fevers? | YES/NO |
| Unexplained lung damage on lung photos or CT scan? | YES/NO |

**Supplementary table 2. Questionnaire results of the non-affected family members. Family history negative (FH-) versus family history positive (FH+)**

|  | **FH-** | **FH+** | **p value** |
| --- | --- | --- | --- |
|  | 28 | 13 |  |
| At least one item answered with ‘Yes’ | 9 (32.1) | 8 (61.5) | 0.15 |
| Recurrent (probable) bacterial infections? – Yes: n (%) | 3 (10.7) | 2 (15.4) | 1.0 |
| > 8 upper respiratory tract infections per year? – Yes: n (%) | 3 (10.7) | 2 (15.4) | 1.0 |
| >2 pneumonias per year? – Yes: n (%) | 1 (3.7) | 0 (0.0) | 1.0 |
| >1 serious invasive infection like meningitis, osteomyelitis (bone infection), pneumonia, sepsis? – Yes: n (%) | 0 (0.0) | 2 (15.4) | 0.19 |
| Infections with atypical presentation, who are unusually serious, or do not respond to treatment? – Yes: n (%) | 1 (3.7) | 1 (7.7) | 1.0 |
| Using antibiotics for >2 months with (almost) no response? – Yes: n (%) | 0 (0.0) | 0 (0.0) | NA |
| Need of IV antibiotics to treat an infection? – Yes: n (%) | 0 (0.0) | 0 (0.0) | NA |
| Abscesses of internal organs? – Yes: n (%) | 0 (0.0) | 1 (7.7) | 0.71 |
| Repeated abscesses of the skin? – Yes: n (%) | 0 (0.0) | 0 (0.0) | NA |
| Frequent yeast infections in mouth or mucous membranes that are hard to cure or recur quickly after stopping treatment? – Yes: n (%) | 2 (7.4) | 0 (0.0) | 0.82 |
| Chronical or recurring episodes of diarrhea? – Yes: n (%) | 0 (0.0) | 1 (7.7) | 0.71 |
| Poor weight evolution, unexplained weight loss or delayed growth? – Yes: n (%) | 0 (0.0) | 0 (0.0) | NA |
| Family members with similar symptoms? – Yes: n (%) | 1 (3.7) | 3 (23.1) | 0.18 |
| Autoimmunity like thyroid disease, diabetes, autoimmune cytopenia, low blood cell counts, bowel diseases? – Yes: n (%) | 3 (10.7) | 3 (23.1) | 0.57 |
| Complications after a vaccination? – Yes: n (%) | 0 (0.0) | 0 (0.0) | NA |
| Did a doctor ever found granulomas? – Yes: n (%) | 1 (3.7) | 0 (0.0) | 1.0 |
| Recurrent unexplained fevers? – Yes: n (%) | 0 (0.0) | 1 (7.7) | 0.71 |
| Unexplained lung damage on lung photos or CT scan? – Yes: n (%) | 0 (0.0) | 0 (0.0) | NA |

**Supplementary table 3-9. Characteristics and antibody levels in serum, saliva and mucosal lining fluid (MLF) of all families.** Families 3-6 are family history negative families. Families 7-9 are family history positive families. URTI = upper respiratory tract infection. DM = Diabetes mellitus. All serum antibodies were measured in the serum collected from the finger prick blood during the study. ^ insufficient material for analysis, ^+^ no sample received

| **Family 3** | **Index (girl)** | **Father** | **Mother** |
| --- | --- | --- | --- |
| **Characteristics** | | | |
| **Age (years)** | 15 | 59 | 50 |
| **Diagnosis** | CVID | - | - |
| **Therapy** | IVIG | - | - |
| **Questionnaire** |  | Granulomas | >8 URTIs; recurrent infections; diarrhea; auto-immunity |
| **Antibody levels (ng/ml)** | | | |
| **Serum IgG** | 1.07E+07 | 1.18E+07 | 4.27E+06 |
| **Serum IgA** | 4.97E+05 | 2.12E+06 | 2.83E+06 |
| **Saliva IgG/IgA** | Missing^ | Missing^ | Missing ^ |
| **MLF IgG** | 5.22E+04 | 1.64E+04 | 1.05E+04 |
| **MLF IgA** | 5.93E+05 | 1.37E+05 | 3.57E+05 |

| **Family 4** | **Index**  **(girl)** | **Mother** | **Father** | **Grandmother (m)** | **Grandfather (m)** | **Grandfather (f)** | **Grandmother (f)** |
| --- | --- | --- | --- | --- | --- | --- | --- |
| **Characteristics** | | | | | | | |
| **Age (years)** | 11 | 39 | 41 | 65 | 69 | 75 | 75 |
| **Diagnosis** | CVID | - | - | - | - | - | - |
| **Therapy** |  | - | - | - | - | - | - |
| **Questionnaire** | - | History of recurrent infections and frequent yeast infections | - |  |  |  | Fistula and infections after breast surgery; thyroid disease |
| **Antibody levels (ng/ml)** | | | | | | | |
| **Serum IgG** | 9.49E+06 | 1.29E+07 | 1.05E+07 | 1.34E+07 | 1.02E+07 | Missing^+^ | 1.02E+07 |
| **Serum IgA** | 9.37E+05 | 6.60E+05 | 2.68E+06 | 2.01E+06 | 2.42E+06 | Missing^+^ | 3.68E+06 |
| **Saliva IgG** | 1.16E+05 | 1.14E+05 | 3.84E+04 | 1.10E+05 | 3.52E+04 | Missing^+^ | Missing^ |
| **Saliva IgA** | 7.12E+06 | 2.50E+06 | 1.42E+06 | 2.70E+06 | 9.28E+05 | Missing^+^ | Missing^ |
| **MLF IgG** | 1.77E+05 | 1.52E+04 | 5.11E+02 | 1.52E+04 | 7.36E+03 | Missing^+^ | 3.95E+04 |
| **MLF IgA** | 2.14E+06 | 1.72E+05 | 3.25E+05 | 2.06E+05 | 1.93E+05 | Missing^+^ | 3.35E+05 |

| **Family 5** | **Index**  **(girl)** | **Father** | **Mother** | **Grand-mother (f)** | **Grand-father (f)** | **Grand-father (m)** | **Grand-mother (m)** |
| --- | --- | --- | --- | --- | --- | --- | --- |
| **Characteristics** | | | | | | | |
| **Age (years)** | 9 | 40 | 33 | 76 | 75 | 61 | 57 |
| **Diagnosis** | CVID | - | - | - | - | - | - |
| **Therapy** | SCIG | - | - | - | - | - | - |
| **Questionnaire** | - | - | - | - | - | - | - |
| **Antibody levels (ng/ml)** | | | | | | | |
| **Serum IgG** | 1.02E+07 | 1.22E+07 | 1.83E+07 | 1.36E+07 | 9.40E+06 | 9.58E+06 | 1.48E+07 |
| **Serum IgA** | 3.68E+06 | 2.25E+05 | 4.47E+06 | 6.60E+05 | 3.45E+06 | 1.48E+06 | 1.03E+06 |
| **Saliva IgG** | Missing^ | Missing^ | 1.40E+04 | 1.26E+04 | Missing^ | Missing^ | 2.43E+04 |
| **Saliva IgA** | Missing^ | Missing^ | 5.57E+05 | 3.90E+05 | Missing^ | Missing^ | 1.31E+06 |
| **MLF IgG** | 3.95E+04 | 3.61E+04 | 3.97E+04 | 4.39E+04 | 9.45E+03 | 2.99E+02 | 3.17E+04 |
| **MLF IgA** | 3.35E+05 | 5.37E+05 | 2.77E+05 | 2.05E+05 | 1.97E+05 | 2.55E+04 | 3.50E+05 |

| **Family 6** | **Index**  **(girl)** | **Father** | **Mother** | **Grand-father (m)** | **Grand-mother (m)** | **Grand-mother (f)** | **Grand-father (f)** |
| --- | --- | --- | --- | --- | --- | --- | --- |
| **Characteristics** | | | | | | | |
| **Age (years)** | 9 | 46 | 43 | 66 | 67 | 74 | 73 |
| **Diagnosis** | CVID | - | - | - | - | - | - |
| **Therapy** | SCIG | - | - | - | - | - | - |
| **Questionnaire** | - | - | Recurrent infections; >8 URTIs; >2 pneumonia | - | - | - | - |
| **Antibody levels (ng/ml)** | | | | | | | |
| **Serum IgG** | 1.01E+07 | 1.60E+07 | 1.12E+07 | 9.56E+06 | 1.24E+07 | 8.48E+06 | 1.45E+07 |
| **Serum IgA** | 5.73E+05 | 4.41E+06 | 2.38E+06 | 3.05E+06 | 1.39E+06 | 2.52E+06 | 2.97E+06 |
| **Saliva IgG** | 2.56E+04 | Missing^ | 7.18E+04 | 3.48E+04 | 1.73E+05 | Missing^ | 6.24E+04 |
| **Saliva IgA** | 5.78E+05 | Missing^ | 9.79E+05 | 8.28E+05 | 3.65E+06 | Missing^ | 2.82E+06 |
| **MLF IgG** | 1.25E+05 | 7.12E+04 | 2.28E+04 | 1.43E+04 | 1.97E+05 | 5.20E+04 | 4.49E+04 |
| **MLF IgA** | 6.55E+05 | 4.04E+05 | 3.84E+05 | 3.00E+05 | 5.34E+05 | 3.14E+05 | 3.07E+05 |

| **Family 7** | **Index**  **(girl)** | **Father** | **Mother** | **Aunt (m)** | **Grandmother (m)** | **Grandfather (m)** | **Grandmother (f)** |
| --- | --- | --- | --- | --- | --- | --- | --- |
| **Characteristics** | | | | | | | |
| **Age (years)** | 6 | 45 | 39 | 42 | 66 | 67 | 77 |
| **Diagnosis** | CVID | - | CVID | - | - | - | - |
| **Therapy** | SCIG | - | IVIG | - | - | - | - |
| **Questionnaire** | - | Recurrent infections; >1 invasive infection | - | - | >1 invasive infection; atypical infection; thyroid disease | Thyroid disease | - |
| **Antibody levels (ng/ml)** | | | | | | | |
| **Serum IgG** | Missing^ | 1.02E+07 | 1.29E+07 | 1.13E+07 | 1.26E+07 | 1.31E+07 | 1.13E+07 |
| **Serum IgA** | Missing^ | 1.60E+06 | 2.81E+05 | 1.63E+05 | 5.58E+05 | 5.22E+05 | 1.12E+06 |
| **Saliva IgG** | 1.41E+04 | 1.49E+04 | 2.48E+04 | Missing^ | 2.83E+05 | 8.11E+04 | 1.99E+05 |
| **Saliva IgA** | 4.08E+05 | 8.49E+05 | 3.02E+05 | Missing^ | 2.06E+06 | 1.30E+06 | 5.28E+06 |
| **MLF IgG** | 1.48E+05 | 4.14E+04 | 3.76E+04 | 7.38E+03 | 1.89E+05 | 1.11E+04 | 2.57E+04 |
| **MLF IgA** | 7.84E+05 | 6.08E+05 | 1.70E+05 | 1.39E+05 | 7.13E+05 | 1.14E+05 | 2.33E+05 |

| **Family 8** | **Index**  **(boy)** | **Brother** | **Father** | **Mother** | **Grand-mother (f)** | **Grand-father (f)** | **Grand-mother (m)** |
| --- | --- | --- | --- | --- | --- | --- | --- |
| **Characteristics** | | | | | | | |
| **Age (years)** | 4 | 5 | 36 | 36 | 67 | 70 | 66 |
| **Diagnosis** | CVID | CVID | - | - | - | - | - |
| **Therapy** | SCIG | SCIG | - | - | - | - | - |
| **Questionnaire** | - | - | - | >8 URTIs; unexplained fever | DM type 2; diarrhea | Abscess of internal organ (appendix) | Recurrent infections; >8 URTIs |
| **Antibody levels (ng/ml)** | | | | | | | |
| **Serum IgG** | 1.15E+07 | 1.10E+07 | 1.34E+07 | 1.22E+07 | 1.03E+07 | 1.73E+07 | 7.38E+06 |
| **Serum IgA** | 6.98E+05 | 3.43E+05 | 2.03E+06 | 1.61E+06 | 3.46E+06 | 1.93E+06 | 1.02E+06 |
| **Saliva IgG** | 4.11E+04 | 6.97E+04 | 2.64E+04 | Missing^ | 3.82E+04 | 5.96E+04 | Missing^ |
| **Saliva IgA** | 1.34E+06 | 1.71E+06 | 1.06E+06 | Missing^ | 4.43E+06 | 2.60E+06 | Missing^ |
| **MLF IgG** | 2.62E+02 | 3.20E+04 | 5.39E+04 | 5.70E+04 | 2.47E+04 | 5.24E+04 | 4.76E+03 |
| **MLF IgA** | 9.91E+03 | 1.89E+05 | 8.41E+05 | 8.55E+05 | 9.25E+05 | 4.86E+05 | 5.39E+05 |

| **Family 9** | **Index (girl)** | **Sister** | **Brother** | **Father** | **Mother** |
| --- | --- | --- | --- | --- | --- |
| **Characteristics** | | | | | |
| **Age (years)** | 22 | 19 | 17 | 56 | 52 |
| **Diagnosis** | CVID | CVID | CVID | - | IgG1 and IgG2 subclass deficiency |
| **Therapy** | IVIG | IVIG | IVIG | - | IVIG |
| **Questionnaire** | - | - | - | - | - |
| **Antibody levels (ng/ml)** | | | | | |
| **Serum IgG** | Missing^+^ | Missing^+^ | 8.78E+06 | 1.00E+07 | 7.20E+06 |
| **Serum IgA** | Missing^+^ | Missing^+^ | 9.76E+05 | 1.13E+06 | 7.28E+05 |
| **Saliva IgG** | Missing^+^ | Missing^+^ | Missing^ | 1.77E+05 | Missing^ |
| **Saliva IgA** | Missing^+^ | Missing^+^ | Missing^ | 4.42E+06 | Missing^ |
| **MLF IgG** | Missing^+^ | Missing^+^ | 4.40E+04 | 1.79E+04 | 3.64E+04 |
| **MLF IgA** | Missing^+^ | Missing^+^ | 3.67E+05 | 7.68E+05 | 9.17E+04 |

**Supplementary table 9: Correlations of IgA+ cells and systemic and mucosal IgA antibody levels in non-affected family members of family history negative families.** Data shows the Spearman correlation coefficient (ρ) and p value (p).

|  | **MBC IgA+** | **PC IgA +** | **Serum IgA** | **MLF IgA** | **Saliva IgA** |
| --- | --- | --- | --- | --- | --- |
| **MBC IgA+** |  |  |  |  |  |
| **PC IgA +** | ρ = 0.411;  p = 0.041 |  |  |  |  |
| **Serum IgA** | ρ = -0.071;  p = 0.747 | ρ = 0.217;  p = 0.319 |  |  |  |
| **MLF IgA** | ρ = 0.237;  p = 0.275 | ρ = 0.155;  p = 0.479 | ρ = 0.276;  p = 0.181 |  |  |
| **Saliva IgA** | ρ = 0.225;  p = 0.419 | ρ = 0.104;  p = 0.714 | ρ = -0.250;  p = 0.368 | ρ = 0.329;  p = 0.232 |  |

**Supplementary table 10: Correlations of IgA+ cells and systemic and mucosal IgA antibody levels in non-affected family members of family history positive families.** Data shows the Spearman correlation coefficient (ρ) and p value (p).

|  | **MBC IgA+** | **PC IgA +** | **Serum IgA** | **MLF IgA** | **Saliva IgA** |
| --- | --- | --- | --- | --- | --- |
| **MBC IgA+** |  |  |  |  |  |
| **PC IgA +** | ρ = 0.231;  p = 0.426 |  |  |  |  |
| **Serum IgA** | ρ = -0.402;  p = 0.155 | ρ = 0.029;  p = 0.928 |  |  |  |
| **MLF IgA** | ρ = 0.090;  p = 0.762 | ρ = 0.073;  p = 0.808 | ρ = 0.349;  p = 0.221 |  |  |
| **Saliva IgA** | ρ = 0.095;  p = 0.840 | ρ = 0.071;  p = 0.882 | ρ = 0.048;  p = 0.935 | ρ = 0.048; p = 0.935 |  |

**Supplementary table 11: Correlations of IgG+ cells and systemic and mucosal IgG antibody levels in non-affected family members of family history negative families.** Data shows the Spearman correlation coefficient (ρ) and p value (p).

|  | **MBC IgG+** | **PC IgG +** | **Serum IgG** | **MLF IgG** | **Saliva IgG** |
| --- | --- | --- | --- | --- | --- |
| **MBC IgG+** |  |  |  |  |  |
| **PC IgG +** | ρ = 0.447; p = 0.025 |  |  |  |  |
| **Serum IgG** | ρ = 0.328; p = 0.127 | ρ = 0.599;  p = 0.003 |  |  |  |
| **MLF IgG** | ρ = 0.410; p = 0.053 | ρ = -0.016;  p = 0.943 | ρ = 0.078;  p = 0.711 |  |  |
| **Saliva IgG** | ρ = -0.364; p = 0.182 | ρ = -0.500;  p = 0.060 | ρ = -0.446;  p = 0.097 | ρ = -0.275;  p = 0.320 |  |

**Supplementary table 12: Correlations of IgG+ cells and systemic and mucosal IgG antibody levels in non-affected family members of family history positive families.** Data shows the Spearman correlation coefficient (ρ) and p value (p).

|  | **MBC IgG+** | **PC IgG +** | **Serum IgG** | **MLF IgG** | **Saliva IgG** |
| --- | --- | --- | --- | --- | --- |
| **MBC IgG+** |  |  |  |  |  |
| **PC IgG +** | ρ = 0.174; p = 0.55 |  |  |  |  |
| **Serum IgG** | ρ = 0.266; p = 0.18 | ρ = 0.183;  p = 0.53 |  |  |  |
| **MLF IgG** | ρ = 0.147; p = 0.62 | ρ = 0.157;  p = 0.59 | ρ = 0.644;  p = 0.015 |  |  |
| **Saliva IgG** | ρ = 0.143; p = 0.75 | ρ = 0.317;  p = 0.44 | ρ = -0.024;  p = 0.98 | ρ = -0.024;  p = 0.98 |  |

**Supplementary table 13: Correlations of IgA+ cells and systemic and mucosal IgA antibody levels in CVID patients (n=13).** Data shows the Spearman correlation coefficient (ρ) and p value (p).

|  | **MBC IgA+** | **PC IgA +** | **Serum IgA** | **MLF IgA** | **Saliva IgA** |
| --- | --- | --- | --- | --- | --- |
| **MBC IgA+** |  |  |  |  |  |
| **PC IgA +** | ρ = 0.379;  p = 0.20 |  |  |  |  |
| **Serum IgA** | ρ = 0.018;  p = 0.97 | ρ = 0.224;  p = 0.54 |  |  |  |
| **MLF IgA** | ρ = -0.100;  p = 0.78 | ρ = 0.155;  p = 0.65 | ρ = 0.151;  p = 0.68 |  |  |
| **Saliva IgA** | ρ = -0.714;  p = 0.088 | ρ = -0.536;  p = 0.24 | ρ = 0.714;  p = 0.14 | ρ = 0.179;  p = 0.71 |  |

**Supplementary table 14: Correlations of IgG+ cells and systemic and mucosal IgG antibody levels in CVID patients (n=13).** Data shows the Spearman correlation coefficient (ρ) and p value (p).

|  | **MBC IgG+** | **PC IgG +** | **Serum IgG** | **MLF IgG** | **Saliva IgG** |
| --- | --- | --- | --- | --- | --- |
| **MBC IgG+** |  |  |  |  |  |
| **PC IgG +** | ρ = 0.709;  p < 0.01 |  |  |  |  |
| **Serum IgG** | ρ = 0.503;  p = 0.14 | ρ = 0.442;  p = 0.20 |  |  |  |
| **MLF IgG** | ρ = -0.309, p = 0.36 | ρ = 0.036;  p = 0.92 | ρ = -0.309;  p = 0.39 |  |  |
| **Saliva IgG** | ρ = -0.714; p = 0.088 | ρ = -0.750;  p = 0.066 | ρ = -0.600;  p = 0.24 | ρ = -0.071;  p = 0.91 |  |

**Supplementary table 15. The association of a positive family history, >8 upper respiratory infections (URTIs) per year, and recurrent infections with systemic and mucosal antibody levels, adjusted for age, in non-affected family members.** Results show the beta coefficient with the 95% confidence interval (CI) and p values for serum, mucosal lining fluid (MLF) and saliva IgG and IgA. A significant association was seen for the effect of a positive family history on MLF IgA levels (p=0.013), and recurrent infections on Serum IgG levels (p=0.013).

|  | | **Family history +** | | **>8 URTIs** | | **Recurrent infections** | |
| --- | --- | --- | --- | --- | --- | --- | --- |
|  | | **β (95% CI)** | **P value** | **β (95% CI)** | **P value** | **β (95% CI)** | **P value** |
| **Serum** | **IgG** | 468119 (-1637481 - 2573720) | 0.65 | -3024771 (-6175511 – 125969) | 0.059 | -3540181 (-6272748 – -807614) | 0.013 |
|  | **IgA** | -538271(-1338026 - 261483) | 0.18 | 54014 (-1238163 – 1346190) | 0.93 | -376011 (-1534831 – 782809) | 0.51 |
| **MLF** | **IgG** | 683 (-30942 – 32308) | 0.97 | -18946 (-68259 – 30367) | 0.44 | -24822 (-68878 – 19235) | 0.26 |
|  | **IgA** | 234254 (51844 – 416664) | 0.013 | 122055 (-189451 – 433561) | 0.43 | -33145 (-316627 – 250337) | 0.81 |
| **Saliva** | **IgG** | 22976 (-51859 – 97810) | 0.53 | -680 (-172844 – 171483) | 0.99 | 14468 (-95534 – 124469) | 0.79 |
|  | **IgA** | -132629 (-2297206 – 2031948) | 0.90 | -1705800 (-6572717 – 3161116) | 0.47 | -306117 (-3459743 – 2847510) | 0.84 |
