# Supplementary material for: Familial patterns of immune dysregulation in CVID: insights from B- and T-cell phenotyping and antibody profiling
Source: Front Immunol. 2026 Mar 13;17:1741900. doi: 10.3389/fimmu.2026.1741900 (PMC13021423; doi:10.3389/fimmu.2026.1741900)

Supplementary figure 1

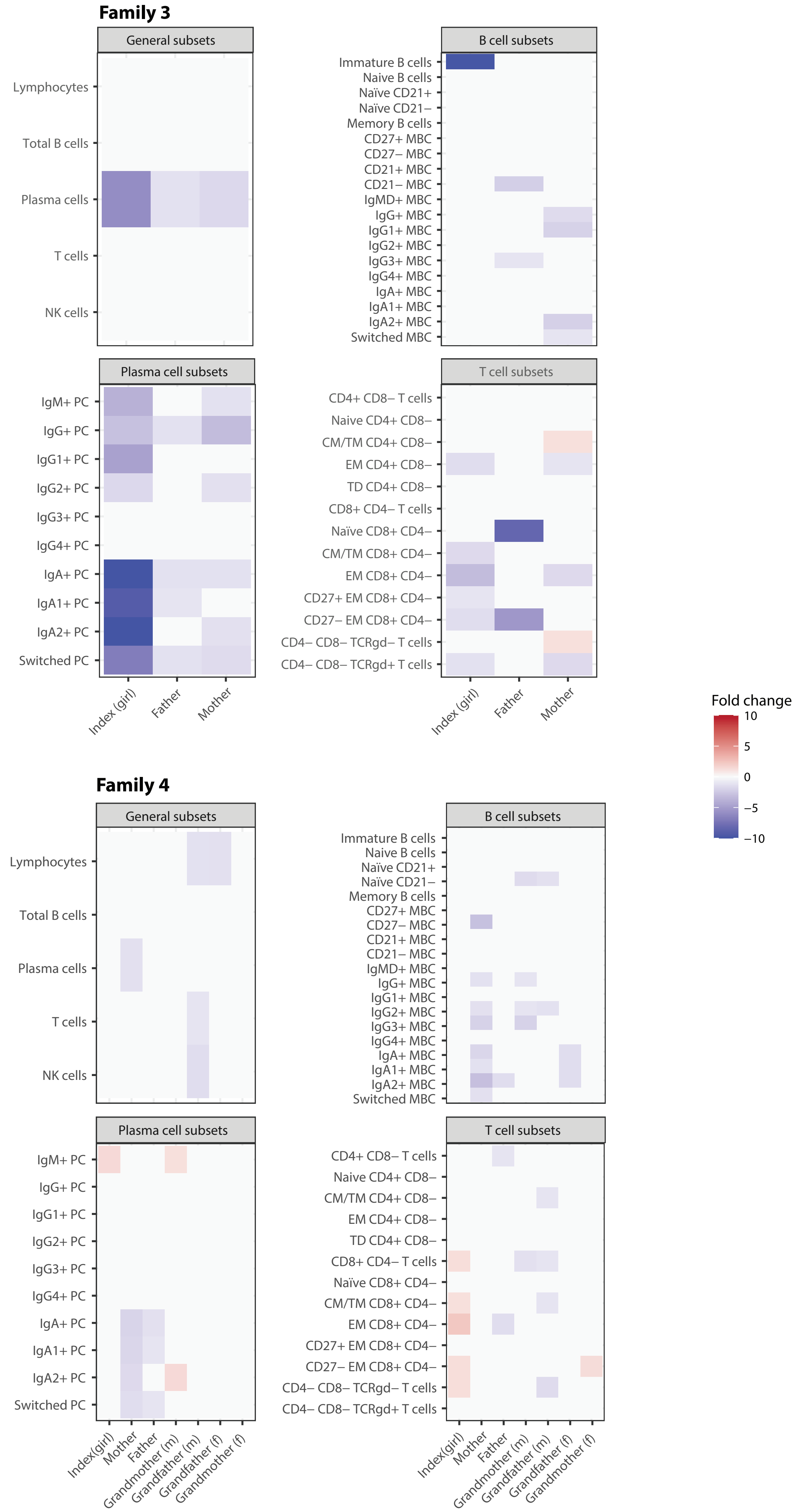

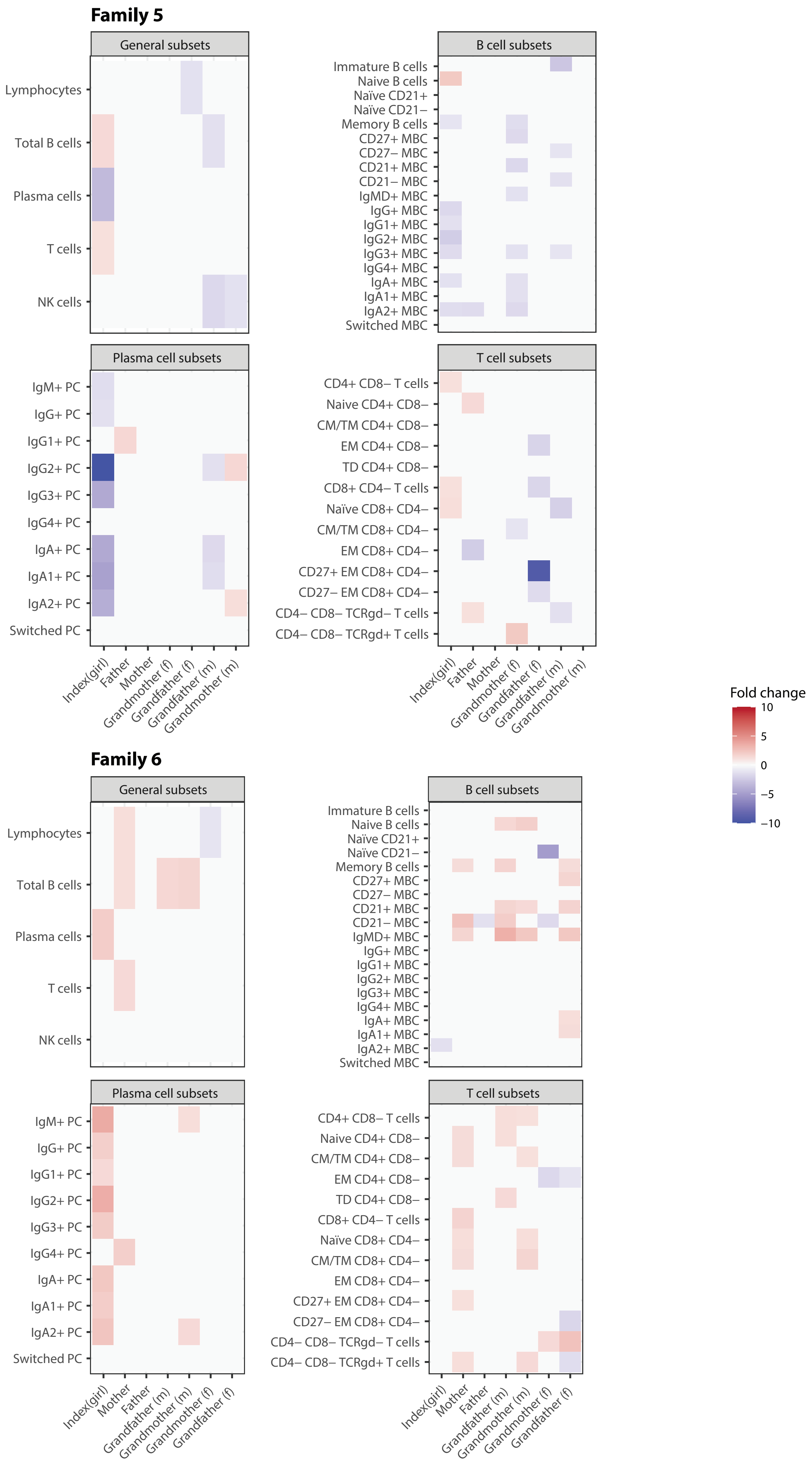
Family 5
General subsets
Lymphocytes
Total B cells
Plasma cells
T cells
NK cells
B cell subsets
Immature B cells
Naive B cells
Naïve CD21+
Naïve CD21–
Memory B cells
CD27+ MBC
CD27– MBC
CD21+ MBC
CD21– MBC
IgMD+ MBC
IgG+ MBC
IgG1+ MBC
IgG2+ MBC
IgG3+ MBC
IgG4+ MBC
IgA+ MBC
IgA1+ MBC
IgA2+ MBC
Switched MBC
Plasma cell subsets
IgM+ PC
IgG+ PC
IgG1+ PC
IgG2+ PC
IgG3+ PC
IgG4+ PC
IgA+ PC
IgA1+ PC
IgA2+ PC
Switched PC
T cell subsets
CD4+ CD8– T cells
Naive CD4+ CD8–
CM/TM CD4+ CD8–
EM CD4+ CD8–
TD CD4+ CD8–
CD8+ CD4– T cells
Naïve CD8+ CD4–
CM/TM CD8+ CD4–
EM CD8+ CD4–
CD27+ EM CD8+ CD4–
CD27– EM CD8+ CD4–
CD4– CD8– TCRgd– T cells
CD4– CD8– TCRgd+ T cells
Index(girl)
Father
Mother
Grandmother (f)
Grandfather (f)
Grandfather (m)
Grandmother (m)
Fold change
10
5
0
−5
−10
Family 6
General subsets
Lymphocytes
Total B cells
Plasma cells
T cells
NK cells
B cell subsets
Immature B cells
Naive B cells
Naïve CD21+
Naïve CD21–
Memory B cells
CD27+ MBC
CD27– MBC
CD21+ MBC
CD21– MBC
IgMD+ MBC
IgG+ MBC
IgG1+ MBC
IgG2+ MBC
IgG3+ MBC
IgG4+ MBC
IgA+ MBC
IgA1+ MBC
IgA2+ MBC
Switched MBC
Plasma cell subsets
IgM+ PC
IgG+ PC
IgG1+ PC
IgG2+ PC
IgG3+ PC
IgG4+ PC
IgA+ PC
IgA1+ PC
IgA2+ PC
Switched PC
T cell subsets
CD4+ CD8– T cells
Naive CD4+ CD8–
CM/TM CD4+ CD8–
EM CD4+ CD8–
TD CD4+ CD8–
CD8+ CD4– T cells
Naïve CD8+ CD4–
CM/TM CD8+ CD4–
EM CD8+ CD4–
CD27+ EM CD8+ CD4–
CD27– EM CD8+ CD4–
CD4– CD8– TCRgd– T cells
CD4– CD8– TCRgd+ T cells
Index(girl)
Mother
Father
Grandfather (m)
Grandmother (m)
Grandmother (f)
Grandfather (f)

Supplementary figure 2

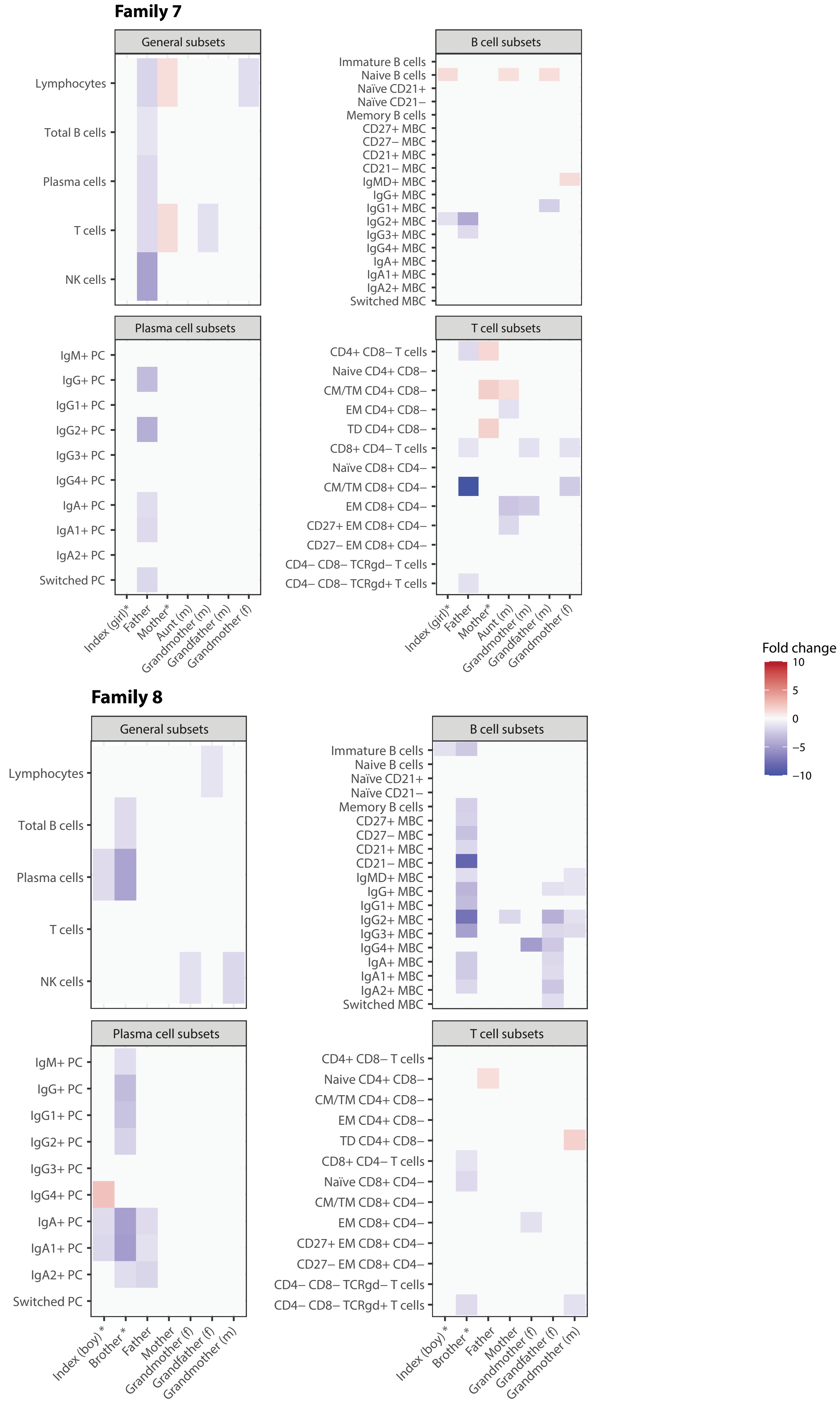

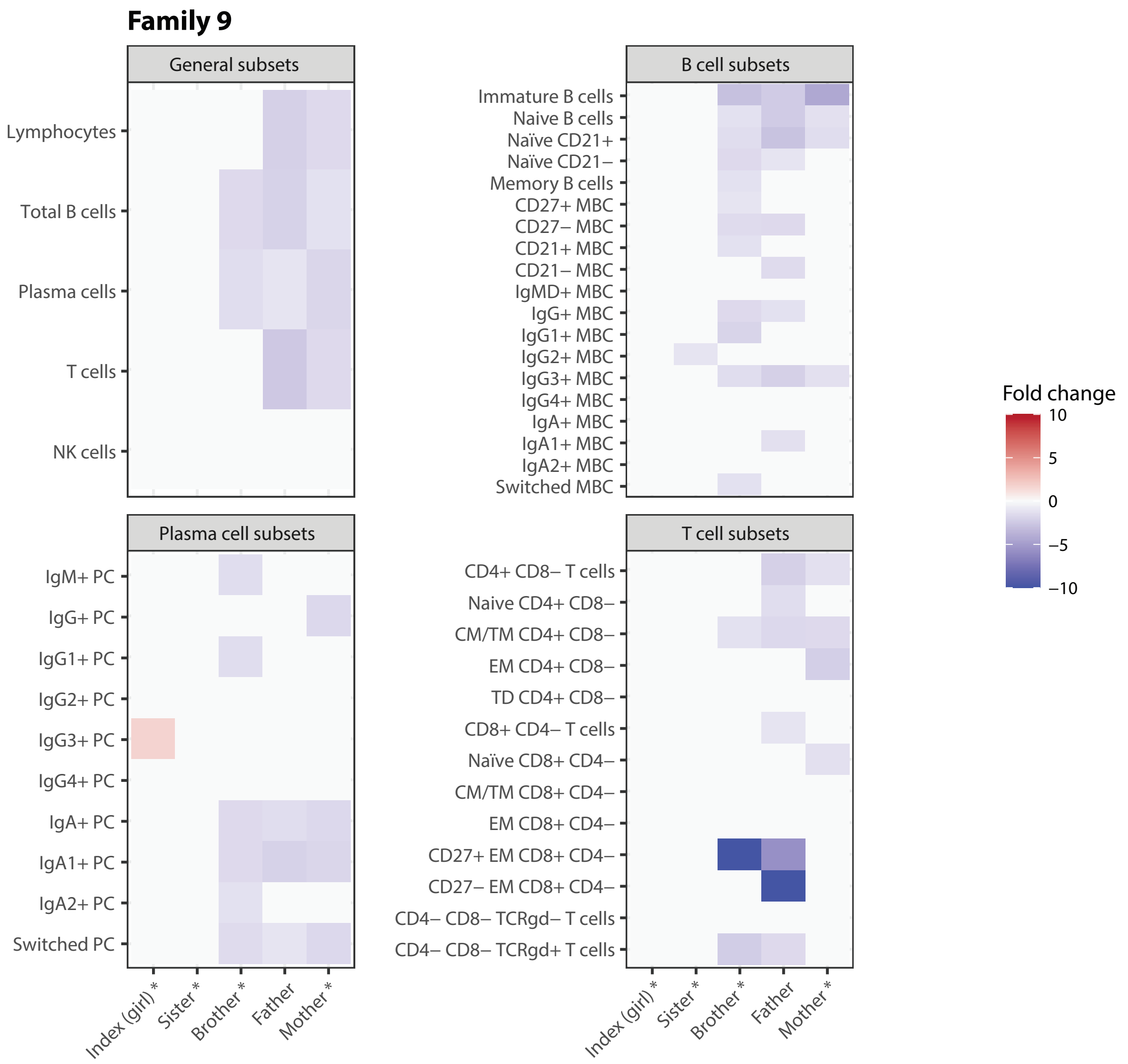
Family 9
General subsets
Lymphocytes
Total B cells
Plasma cells
T cells
NK cells
B cell subsets
Immature B cells
Naive B cells
Naïve CD21+
Naïve CD21–
Memory B cells
CD27+ MBC
CD27– MBC
CD21+ MBC
CD21– MBC
IgMD+ MBC
IgG+ MBC
IgG1+ MBC
IgG2+ MBC
IgG3+ MBC
IgG4+ MBC
IgA+ MBC
IgA1+ MBC
IgA2+ MBC
Switched MBC
Plasma cell subsets
IgM+ PC
IgG+ PC
IgG1+ PC
IgG2+ PC
IgG3+ PC
IgG4+ PC
IgA+ PC
IgA1+ PC
IgA2+ PC
Switched PC
T cell subsets
CD4+ CD8– T cells
Naive CD4+ CD8–
CM/TM CD4+ CD8–
EM CD4+ CD8–
TD CD4+ CD8–
CD8+ CD4– T cells
Naïve CD8+ CD4–
CM/TM CD8+ CD4–
EM CD8+ CD4–
CD27+ EM CD8+ CD4–
CD27– EM CD8+ CD4–
CD4– CD8– TCRgd– T cells
CD4– CD8– TCRgd+ T cells
Index (girl) *
Sister *
Brother *
Father
Mother *
Fold change
10
5
0
–5
–10

Supplementary figure 3

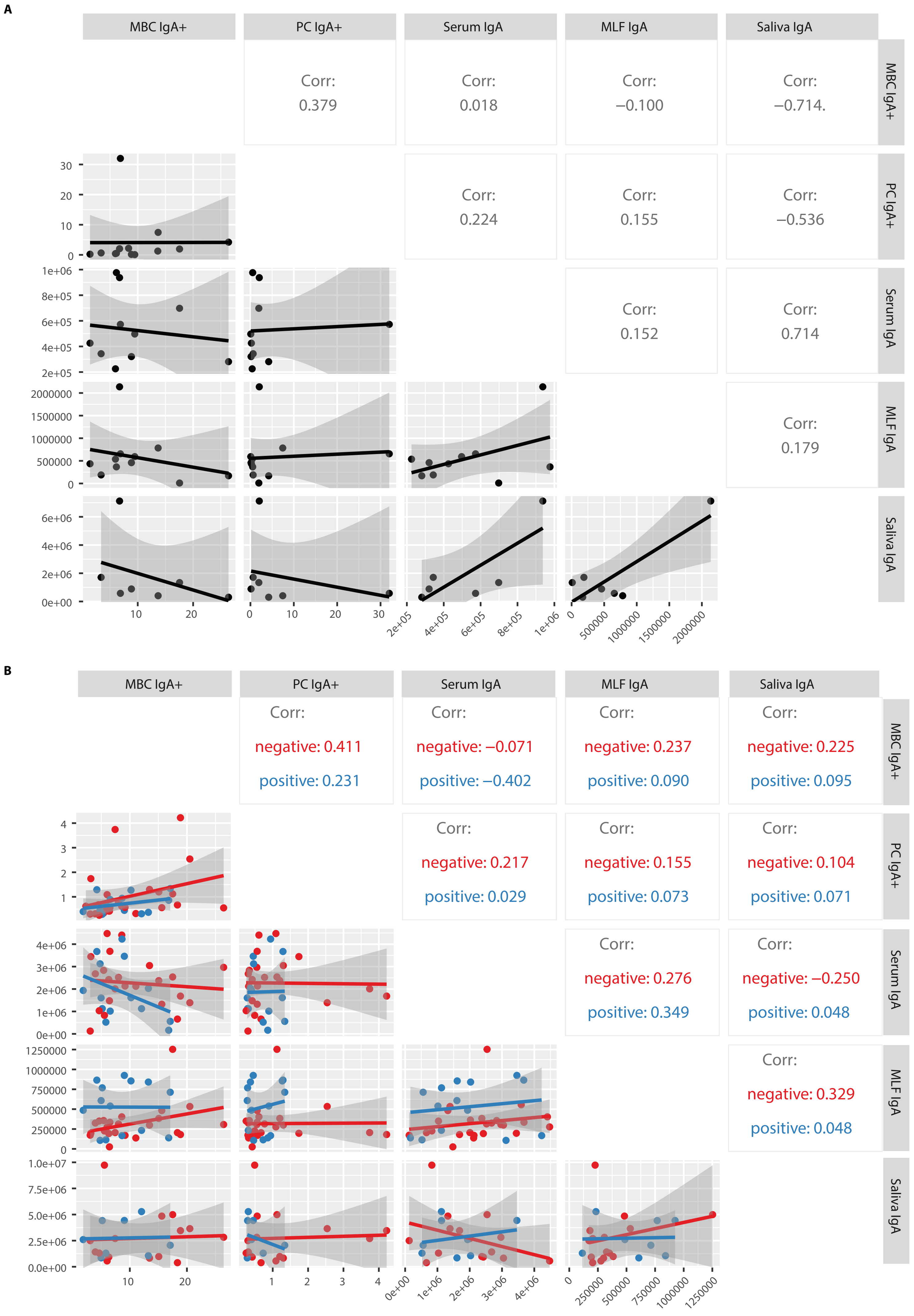

Supplementary figure 4

A

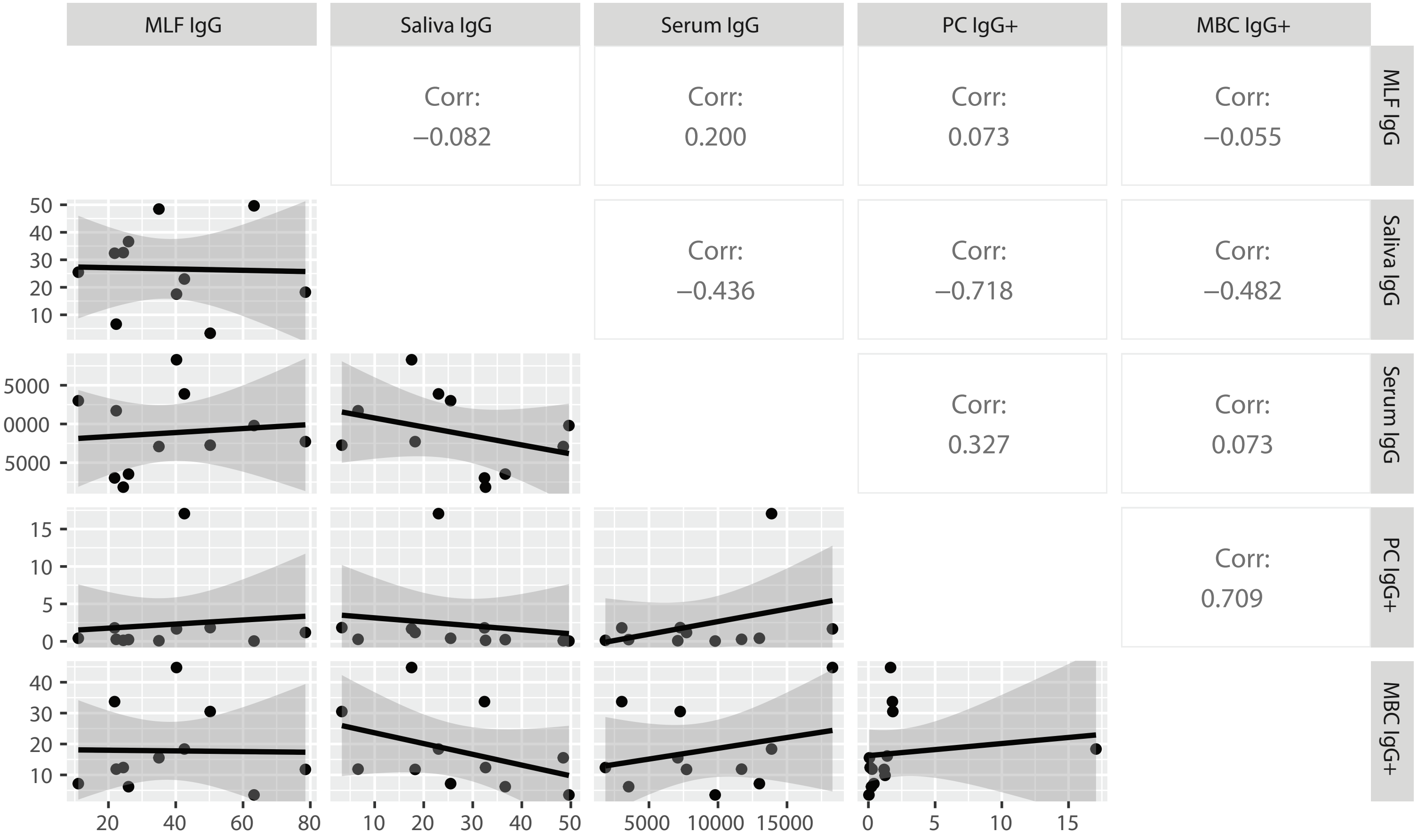

B

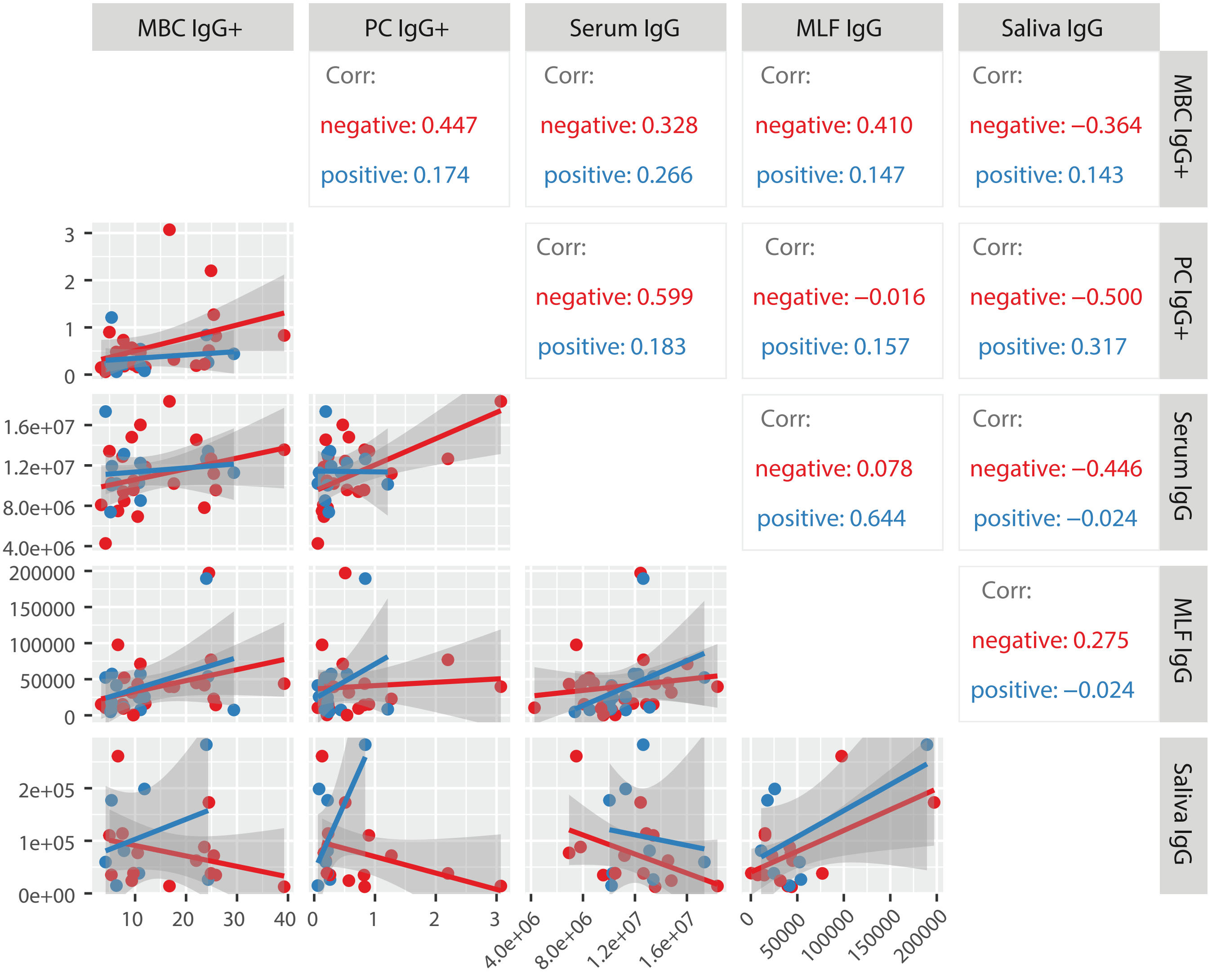

Supplement: Supplementary Figure 1 — Immunophenotyping of families with a negative history for CVID show aberrancies in the B- and T-cell compartment in both the index cases as non-affected family members. Absolute counts were compared to age-specific normal reference values and expressed as a foldchange relative to the 5th and 95th percentile of those reference values. (m) indicates a relative from mothers side, (f) indicates a relative from fathers side. NK, natural killer; PC, plasma cell, MBC, memory B-cell, CM, central memory; TM, transitional memory; EM, effector memory; TD, terminally differentiated. Grey fields indicate that there were no measurable cells present of this cell type. [file DataSheet1.pdf]
